# In vivo adenine base editing corrects newborn murine model of Hurler syndrome

**DOI:** 10.1186/s43556-023-00120-8

**Published:** 2023-02-23

**Authors:** Jing Su, Xiu Jin, Kaiqin She, Yi Liu, Li Song, Qinyu Zhao, Jianlu Xiao, Ruiting Li, Hongxin Deng, Fang Lu, Yang Yang

**Affiliations:** 1 State Key Laboratory of Biotherapy and Cancer Center, West China Hospital, Sichuan University and Collaborative Innovation Center, Ke-yuan Road 4, No. 1, Gao-peng Street, Chengdu, 610041 Sichuan China; 2 Department of Ophthalmology, West China Hospital, Sichuan University, Chengdu, Sichuan China

**Keywords:** Lysosomal storage disease, Mucopolysaccharidosis type I, Monogenic disease, Adeno-associated virus, Base editing

## Abstract

Mucopolysaccharidosis type I (MPS I) is a severe disease caused by loss-of-function mutation variants in the α-L-iduronidase (*Idua*) gene. In vivo genome editing represents a promising strategy to correct *Idua* mutations, and has the potential to permanently restore IDUA function over the lifespan of patients. Here, we used adenine base editing to directly convert A > G (TAG>TGG) in a newborn murine model harboring the *Idua-W392X* mutation, which recapitulates the human condition and is analogous to the highly prevalent human W402X mutation. We engineered a split-intein dual-adeno-associated virus 9 (AAV9) adenine base editor to circumvent the package size limit of AAV vectors. Intravenous injection of the AAV9-base editor system into MPS IH newborn mice led to sustained enzyme expression sufficient for correction of metabolic disease (GAGs substrate accumulation) and prevention of neurobehavioral deficits. We observed a reversion of the W392X mutation in 22.46 ± 6.74% of hepatocytes, 11.18 ± 5.25% of heart and 0.34 ± 0.12% of brain, along with decreased GAGs storage in peripheral organs (liver, spleen, lung and kidney). Collectively, these data showed the promise of a base editing approach to precisely correct a common genetic cause of MPS I in vivo and could be broadly applicable to the treatment of a wide array of monogenic diseases.

**Supplementary Information:**

The online version contains supplementary material available at 10.1186/s43556-023-00120-8.

## Introduction

Mucopolysaccharidosis type I (MPS I) is a severe metabolic disorder caused by deficiency of the lysosomal enzyme, α-L-iduronidase (IDUA), which can catalyze the degradation of glycosaminoglycans (GAGs) heparan and dermatan sulfates. The accumulation of GAGs leads to multi-systemic pathologies and diverse clinical manifestations, including cardiomyopathy, hepatosplenomegaly, upper airway obstruction and progressive neurological disease [1, 2]. According to the severity of the disease, MPS I was classified as mild (Scheie syndrome or MPS IS; MIM#607016), moderate (Hurler-Scheie syndrome or MPS IH/S; MIM#607015) and severe (Hurler syndrome or MPS IH; MIM#607014) subtypes. MPS IH occurs in approximately 1 in 100,000 newborns and is caused by a variation in the *Idua* gene [3–5]. So far, more than 200 pathogenic variants have been reported, including splicing mutations, insertions, deletions, and missense/nonsense mutations [4]. One of the most common mutations (G → A; W402X) accounts for over 40% of patients [6–8].

MPS IH patients begin to show signs of disease within the first 6 months after birth, and will usually die within the first decade without treatment [9]. Therefore, early diagnosis and treatment are essential to prevent the development of serious manifestations. Current approved treatments include enzyme replacement therapy (ERT) and hematopoietic stem cell transplantation (HSCT) [10]. HSCT is considered the standard of care for MPS IH patients, but its success depends on early treatment. Although these treatments can significantly improve disease outcomes and prolong life, there is still a considerable disease burden [11]. Many MPS I-related gene therapies and gene editing approaches are under investigation. Studies have reported that the *Idua* gene was delivered to large animals (dog, cat, and rhesus macaques) by adeno-associated virus (AAV) vector through systemic administration or intrathecal injection, effectively alleviating liver, cardiovascular and brain disease phenotypes [12–14]. In addition, AAV-mediated zinc finger nucleases and proprietary system gene editing also increased the expression of IDUA in vivo and decreased the GAGs storage in MPS I mice (*Idua*^−/−^) [15, 16]. Although some promising gene therapy and gene editing results have been obtained in animal models [17, 18], gene therapy may cause potential loss of an episomal transgene and gene editing may cause unwanted deletion-insertion mutagenesis due to DNA double-strand breaks (DSBs). Base editing can directly convert targeted base pairs without generating DSBs and with minimal indels, so it is considered more suitable for the treatment of human monogenetic inheritance [19, 20]. Adenine base editors (ABEs) which is composed of dCas9 and adenine deaminase can convert A•T to G•C. We reasoned that ABEs can effectively correct MPS IH with G > A point mutation.

In this study, we have developed an AAV9-mediated ABE to directly convert A > G (TAG>TGG) in a newborn murine model harboring the *Idua*-W392X mutation, which recapitulates the human condition and is analogous to the highly prevalent human W402X mutation. We found partial correction of the pathogenic mutation and, biochemical and neurobehavioral deficits in MPS IH mice 12 weeks and 32 weeks after treatment. These findings provide a potential therapeutic approach for MPS IH through direct correction of pathogenic mutations in vivo, informing the application of base editing strategies in the treatment of monogenetic disorders.

## Results

### In vitro screening of the optimal ABE and sgRNA combination targeting the *Idua*-W392X mutation

**Fig. 1:**
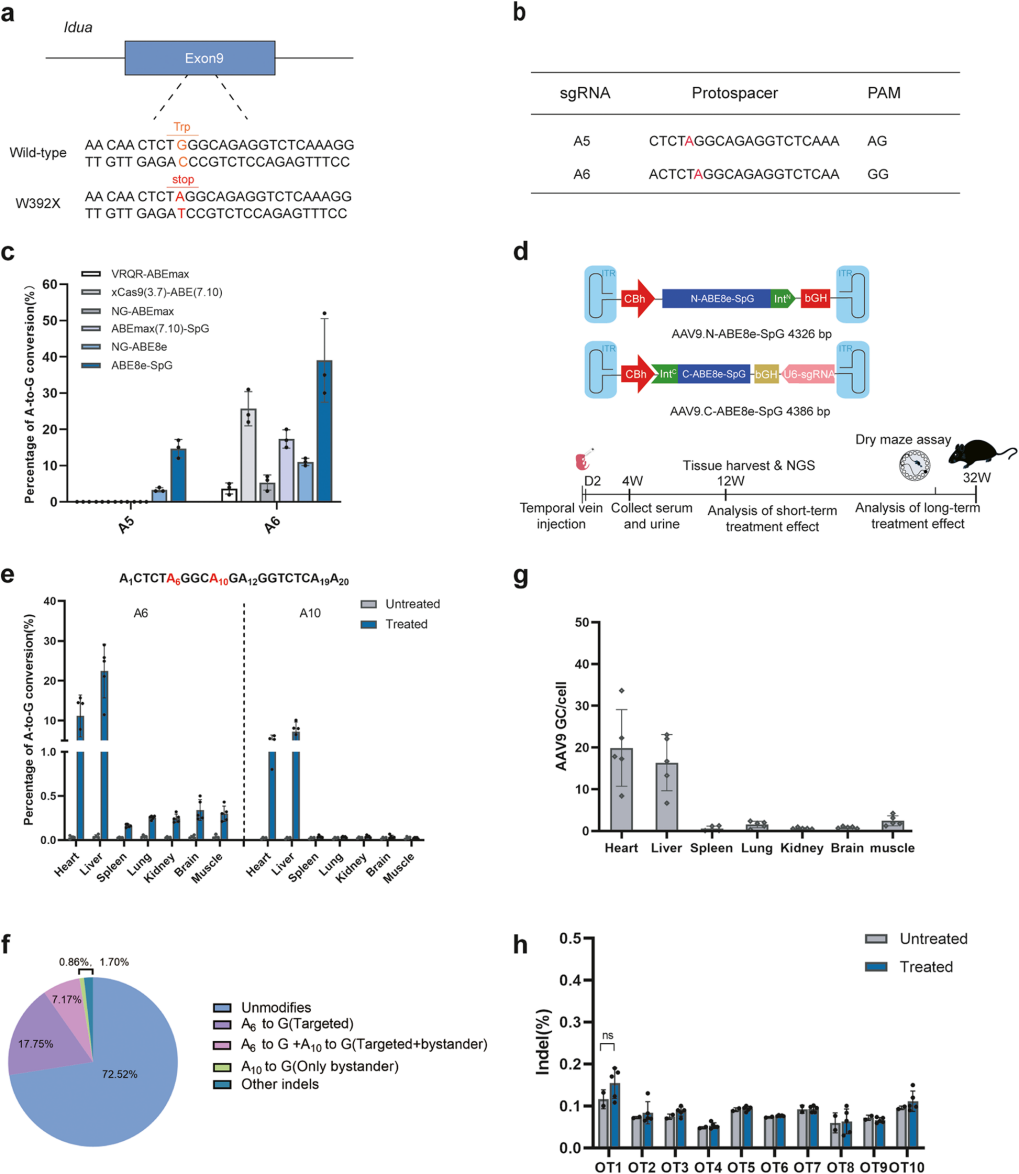
In vitro screening of optimal ABE and sgRNA, and in vivo validation of pathogenic point mutation correction by AAV9-ABE. **a** The *Idua-*W392X mice has a homozygous G•C to A•T nonsense mutation in exon 9 of the *Idua* gene, changing tryptophan (orange) to a stop codon (red). **b** sgRNA-A5 and sgRNA-A6 were designed to target mutation site (red letter) in the editing window of ABEs. **c** Sanger sequencing analysis of correction efficiency of different ABE and sgRNA combinations in mutant cell lines. **d** Schematic diagram of the genomes of two AAV viral vectors encoding split-intein ABE8e-SpG (top) and a summary of the in vivo experiments (bottom). **e** The correction efficiency of pathogenic mutations in mouse tissue genomic DNA was detected by NGS. Untreated MPS IH mice (*n* = 4) were included as control. Treated MPS IH mice (*n* = 5). Mean ± SD are shown. **f** The pie charts show the average composition of allele variants at the DNA levels in liver tissues of a representative treated MPS IH mice. **g** Quantitative analysis of viral genome copy number in various tissues by qPCR. Mean ± SD are shown. **h** NGS analysis of the top 10 potential off-target sites in liver DNA samples. Untreated MPS IH mice (*n* = 2) were included as control. Treated MPS IH mice (*n* = 5). Mean ± SD are shown. One-way ANOVA with Tukey’s post-hoc test, ns = non-significant

*Idua*-W392X mouse is a knock-in disease model of MPS IH that introduces a nucleotide change into the mouse *Idua* gene [21], resulting in a nonsense mutation (G > A) in codon W392 (Fig. 1a). To evaluate the base editing efficiency in vitro, we generated HEK293-*Idua* mutant cell lines by stably integrating the *Idua*-W392X sequence into the AAVS1 genomic locus using CRISPR/Cas9 (Supplemental Fig. 1 and Supplemental Table 1). We first searched for protospacer sequences that span the targeted base. Different ABEs variants have different base editing windows, such as protospacer positions 4-8 for SpABE8e variants, positions 4-7 for ABEmax variants and ABE7.10 variants) [22–24]. Based on the sequence around the targeted site, we designed two sgRNA with NG protospacer-adjacent motif (PAM) suitable for most ABEs (Fig. 1b). VRQR-ABEmax, xCas9(3.7)-ABE(× 7.10), NG-ABEmax, NG-ABE8e and ABEmax(7.10)-SpG are compatible with the PAM sequences of two sgRNAs (Fig. 1b). We also engineered an ABE8e-SpG similar to NG-ABE8e base editors recently described by Richter et al. [23] We co-transfected these adenine base editors with sgRNA-A5 or sgRNA-A6 into HEK293-*Idua* mutant cell lines to screen the most effective base editors 72 h after transfection, respectively*.* Sanger sequencing showed that sgRNA-A6 had higher on-target editing efficiency than sgRNA-A5, and the editing efficiency ranged from 3% to 39% (Fig. 1c). The highest on-target editing efficiency was observed with ABE8e-SpG co-transfected with sgRNA-A6, which was then selected for further studies (Fig. 1c). Then, we engineered a split-intein dual-AAV system to effectively deliver the base editors to mice in vivo, each expressing one half of the base editor (referred to as N-ABE8e.SpG and C-ABE8e.SpG.sgRNA-A6). The in vitro results indicated that the editing efficiency of the split-intein dual-AAV system was lower than that of the full-length ABE8e-SpG version which might be due to a lower abundance of the reconstituted base editor (Supplemental Fig. 2).

### In vivo base editing corrects the W392X mutation in the newborn MPS IH mice

Since MPS IH is a multi-system disease, we chose the AAV9 serotype for its broad tissue tropism to package the split-intein base editor (refer to as AAV9.N-ABE8e-SpG and AAV9.C-ABE8e-SpG) (Fig. 1d) [25, 26]. We performed temporal vein injection with AAV9.N-ABE8e-SpG (3 × 10^11^ GC/mouse) and AAV9.C-ABE8e-SpG (3 × 10^11^ GC/mouse) in newborn *Idua*-W392X mice and evaluated the short- and long-term therapeutic effects after treatment (Fig. 1d). Twelve weeks after AAV9 injection, a subset of the mice were sacrificed, and the editing efficiency was evaluated in various tissues. Next-generation sequencing (NGS) results showed effective correction in heart (11.18 ± 5.25%) and liver (22.46 ± 6.74%), and low-level correction in the spleen (0.17 ± 0.02%), lung (0.25 ± 0.02%), kidney (0.25 ± 0.05%), brain (0.34 ± 0.12%) and muscle (0.30 ± 0.09%) (*n* = 5) (Fig. 1e). In addition to targeted A6 editing, we also detected 4.06 ± 2.30% and 7.26 ± 2.41% of bystander editing at the A10 site in the heart and liver tissues, respectively, resulting in synonymous mutations (GCA to GCG, Ala 393 Ala) (Fig. 1e). When we examined the percentage of precise correction edits without bystander editing using liver tissue as a representative, the correction efficiency was still as high as 17.75%, while bystander editing alone was only 0.86% (Fig. 1f).

Consistent with the NGS results, tissue biodistribution data revealed high AAV9 transduction in the heart (19.89 ± 9.20 GC/cell) and liver (16.35 ± 6.73 GC/cell), with copy numbers below 2.50 GC/cell in other tissues (Fig. 1g). The algorithm described in www.benchling.com identified the top 10 potential off-target sites for sgRNA-A6 (Supplemental Table 2). These off-target sites were amplified by nest PCR from the liver tissue genomic DNA and deep sequenced with NGS (Supplemental Table 3). We observed indel rates in AAV-ABE-treated MPS IH mice similar to those in uninjected-untreated MPS IH mice at these sites, suggesting that sgRNA-A6 specifically targets the intended sites (Fig. 1h).

### In vivo base editing increases IDUA enzyme activity and decreases GAGs storage in MPS IH mice

**Fig. 2:**
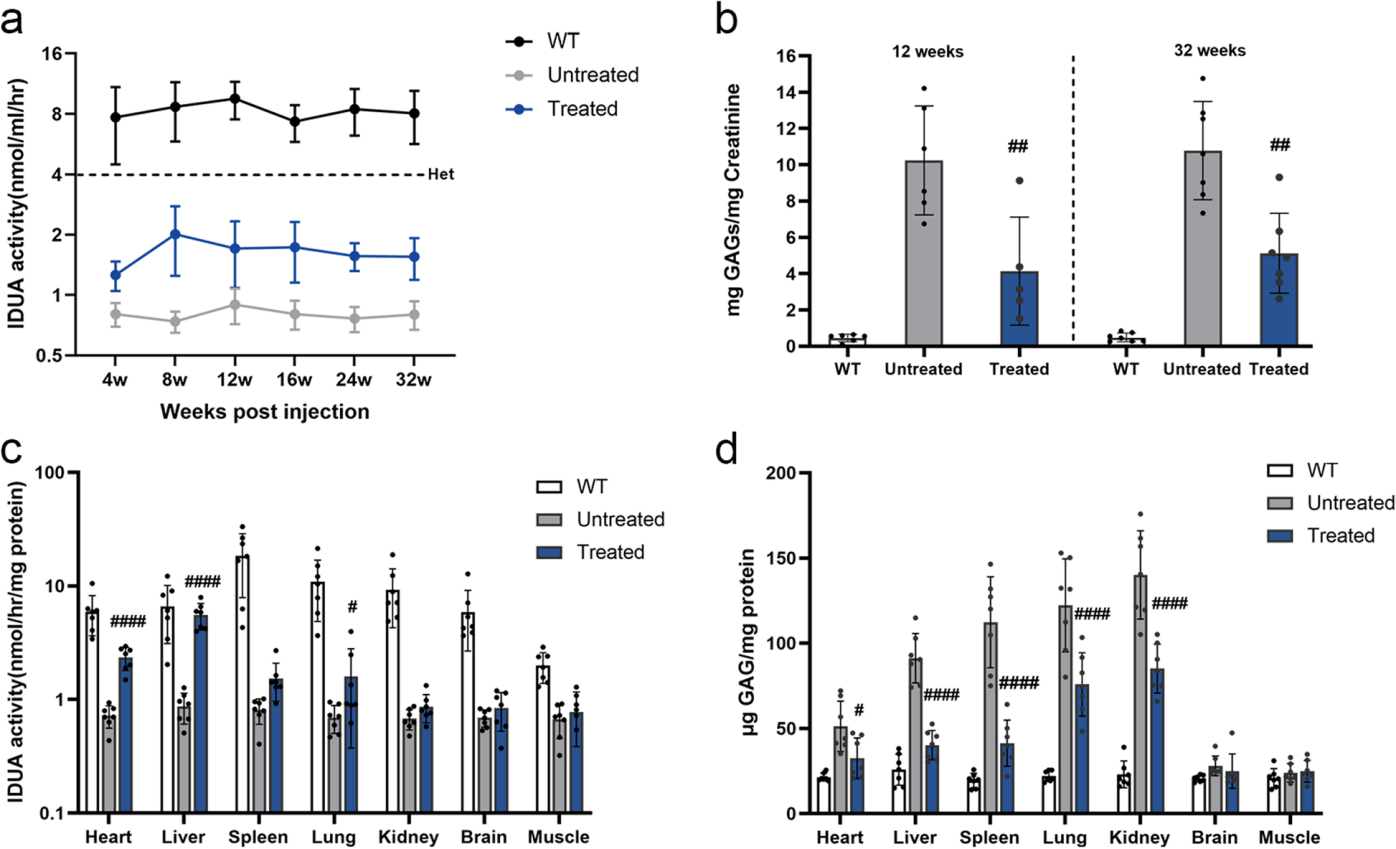
In vivo base editing enables sustained biochemical correction in MPS IH mice. **a** Time course of serum IDUA activity was measured 4 weeks after injection. Dotted line indicates the serum IDUA activity of heterozygous MPS IH mice. Mean ± SD are shown, *n* = 7 per each group. **b** Urine GAGs was detected 12 weeks and 32 weeks after injection. At 12 weeks, WT mice (*n* = 6), untreated MPS IH mice (*n* = 6), treated MPS IH mice (*n* = 5). At 32 weeks, *n* = 7 per each group. Mean ± SD are shown. Comparison between treated and untreated groups, ##*p* < 0.01, one-way ANOVA analysis with Tukey’s post-hoc test. **c** Tissue IDUA activity was detected in various tissues 32 weeks after injection. **d** Tissue GAGs storage was detected in various tissues 32 weeks after injection. (**c, d**) WT mice (*n* = 7) and untreated MPS IH mice (*n* = 7) were included as control. Treated MPS IH mice (*n* = 7). Mean ± SD are shown. The treated MPS IH mice were compared with the untreated MPS IH mice, #*p* < 0.05, ####*p* < 0.0001, one-way ANOVA analysis with Tukey’s post-hoc test

In MPS IH patients and *Idua*-W392X mice, there is almost no IDUA enzyme, and thus GAGs accumulate in urine and tissues [27]. A group of MPS IH mice treated with the AAV9-base editor system were kept for 32 weeks to evaluate the long-term treatment effects. Serum was collected weekly from 4 weeks after injection to evaluate IDUA enzyme activity. We observed that the serum IDUA enzyme activity of the untreated mice was lower than 0.80 nmol/ml/hr., and the serum IDUA enzyme activity of the treated mice was maintained at about 19.80% of wild-type C57BL/6 J (WT) mice (mean of 1.64 and 8.28 nmol/ml/hr., respectively) at multiple time points throughout the study period (Fig. 2a). The urine GAGs level in the treated MPS IH mice was about 59.57% and 52.60% lower than that in the untreated MPS IH mice at 12 weeks and 32 weeks post injection, respectively (12 weeks: 10.24 ± 3.00 vs 4.14 ± 2.98 mg GAGs/mg creatinine; 32 weeks: 10.80 ± 2.70 vs 5.12 ± 2.20 mg GAGs/mg creatinine) (Fig. 2b).

In addition, we observed an increase in IDUA activity and a decrease in GAGs storage in various tissues of 12-week-treated mice compared with untreated mice, especially in heart and liver tissues (Supplemental Fig. 3). MPS IH is a progressive multisystem disease characterized by a continuum of severity [28]. After 32 weeks of treatment, IDUA activity assay results showed that the IDUA activity in the heart, liver and lung was significantly increased in the treated mice, reaching up to 27.35%, 70.87% and 8.25% of the activity in the WT mice, respectively. Slight increases of the IDUA activity in other tissues were also observed, corresponding to about 3.90% (spleen), 2.01% (kidney) and 2.47% (brain) of the activity in the WT mice, respectively, but with no significant difference (Fig. 2c). Furthermore, the GAGs storage in the peripheral tissues of the treated group were significantly reduced compared with that in the untreated MPS I mice, and there was no significant difference of the GAGs storage in the heart and liver between the treated MPS IH mice and the WT mice (Fig. 2d). These results demonstrated that in vivo base editing can sustain long-term biochemical rescue in treated MPS IH mice.

### In vivo base editing improves cardiac function and skeletal abnormalities in MPS IH mice

**Fig. 3:**
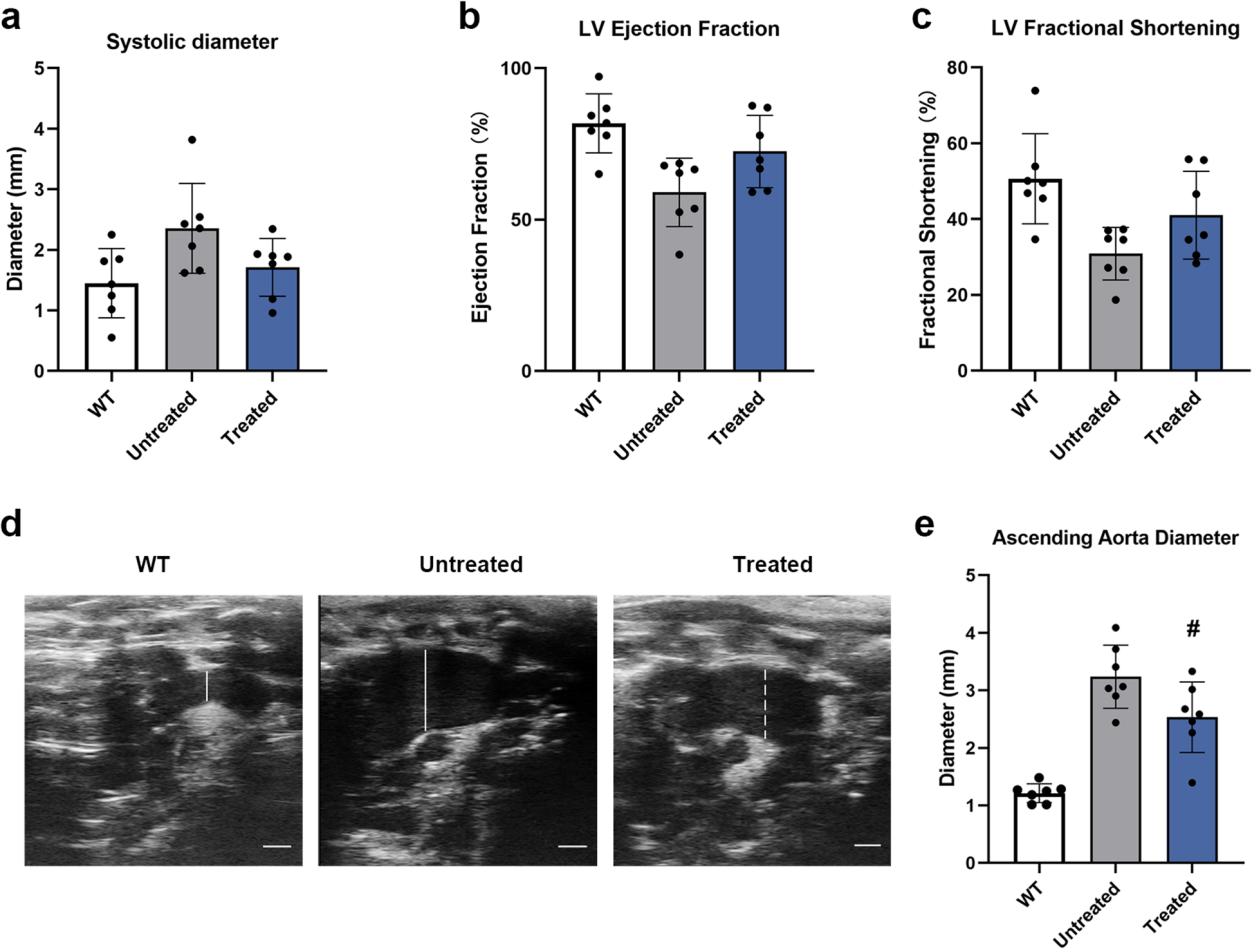
In vivo base editing improves cardiac function in MPS IH mice. **a-c** Echocardiographic parameters (LV systolic diameter, ejection fraction and fractional shortening) were measured at 32 weeks post-injection in WT mice (*n* = 7), untreated MPS IH mice (*n* = 7) and treated MPS I mice (*n* = 7). Mean ± SD are shown. The treated MPS IH mice were compared with the untreated MPS IH mice, one-way ANOVA analysis with Tukey’s post-hoc test. (**a**) *p* = 0.07. (**b**) *p* = 0.0506. (**c**) *p* = 0.07. **d** Representative ultrasound images of the ascending aorta in 32-week-old mice. **e** The results of measuring the ascending aorta diameter of mice (*n* = 7, each group). Comparison between treated and untreated groups, #*p* < 0.05, one-way ANOVA analysis with Tukey’s post-hoc test

Cardiac involvement has been reported in all MPS syndromes, which often manifests as heart valve thickening, abnormal function and aortic dilatation [29–31]. We performed echocardiographic analysis of 32-week-old mice to assess cardiac function. As shown in Fig. 3a-c, the cardiac systolic function and ejection fraction in treated MPS IH mice were higher than those seen in untreated MPS IH mice. Furthermore, the diameter of the ascending aortic arch in the treated MPS IH mice was significantly reduced compared to that in the untreated group (Fig. 3d and e). These data demonstrated that in vivo base editing can partially improve cardiac function and prevent dilation of the aorta in the newborn MPS IH mice.

**Fig. 4:**
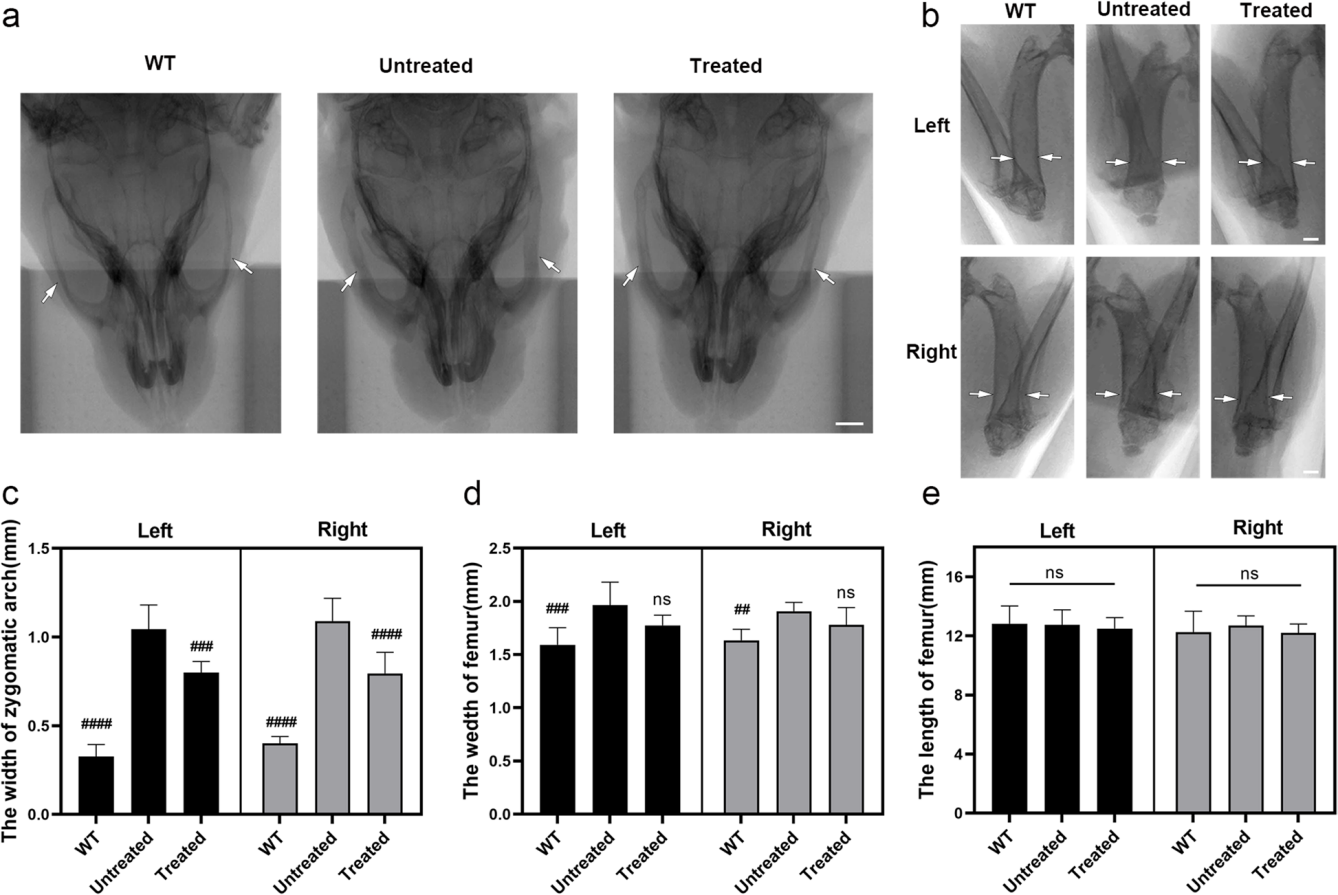
In vivo base editing rescues skeletal abnormalities in MPS IH mice. **a** Representative micro-CT images of 32-week-old mice showing zygomatic arches (white arrows). The zygomatic arch widened significantly in untreated MPS IH mice. Scale bar, 2 mm. **b** Representative micro-CT image of a 32-week-old mouse showing the femur. The two white arrows in the same image indicate the width of the femur. Scale bar, 1 mm. **c-e** Quantification of zygomatic arch width, femur width and femur length. Mean ± SD are shown, *n* = 7 per each group. The WT mice and treated MPS IH mice were compared with the untreated MPS IH mice, ##*p* < 0.01, ###*p* < 0.001, ####*p* < 0.0001, one-way ANOVA analysis with Tukey’s post-hoc test

The skeletal abnormalities of MPS IH are often difficult to treat and severely impact the patient’s quality of life [32]. Skeletal abnormalities in MPS IH mice often include thickening of the zygomatic arch and femur. The microcomputed tomography (micro-CT) scan revealed that the zygomatic arches and femurs of 12-week-old untreated MPS IH mice were not significantly different from those of WT mice and treated MPS IH mice (Supplemental Fig. 4). Skeletal changes in MPS IH mice are progressive. At 32 weeks after treatment, the zygomatic arch of untreated mice was found to be about 1.9-fold wider than that of WT mice, respectively (Fig. 4a and c). This abnormal phenotype was consistent with the coarse facial features of MPS IH individuals [21]. The width of the zygomatic arch in MPS IH treated mice was reduced by 25.3% compared with that in untreated mice (Fig. 4a and c). Similarly, the femur of untreated MPS IH mice was significantly wider than that of WT mice, while the width of treated mice was reduced by 10.6% compared with untreated mice (Fig. 4b and d). As we previously reported [33], we found no difference in femur length between MPS IH mice and age-matched WT mice and treated MPS IH mice (Fig. 4e).

### In vivo base editing reverses lysosomal storage damage in MPS IH mice

**Fig. 5:**
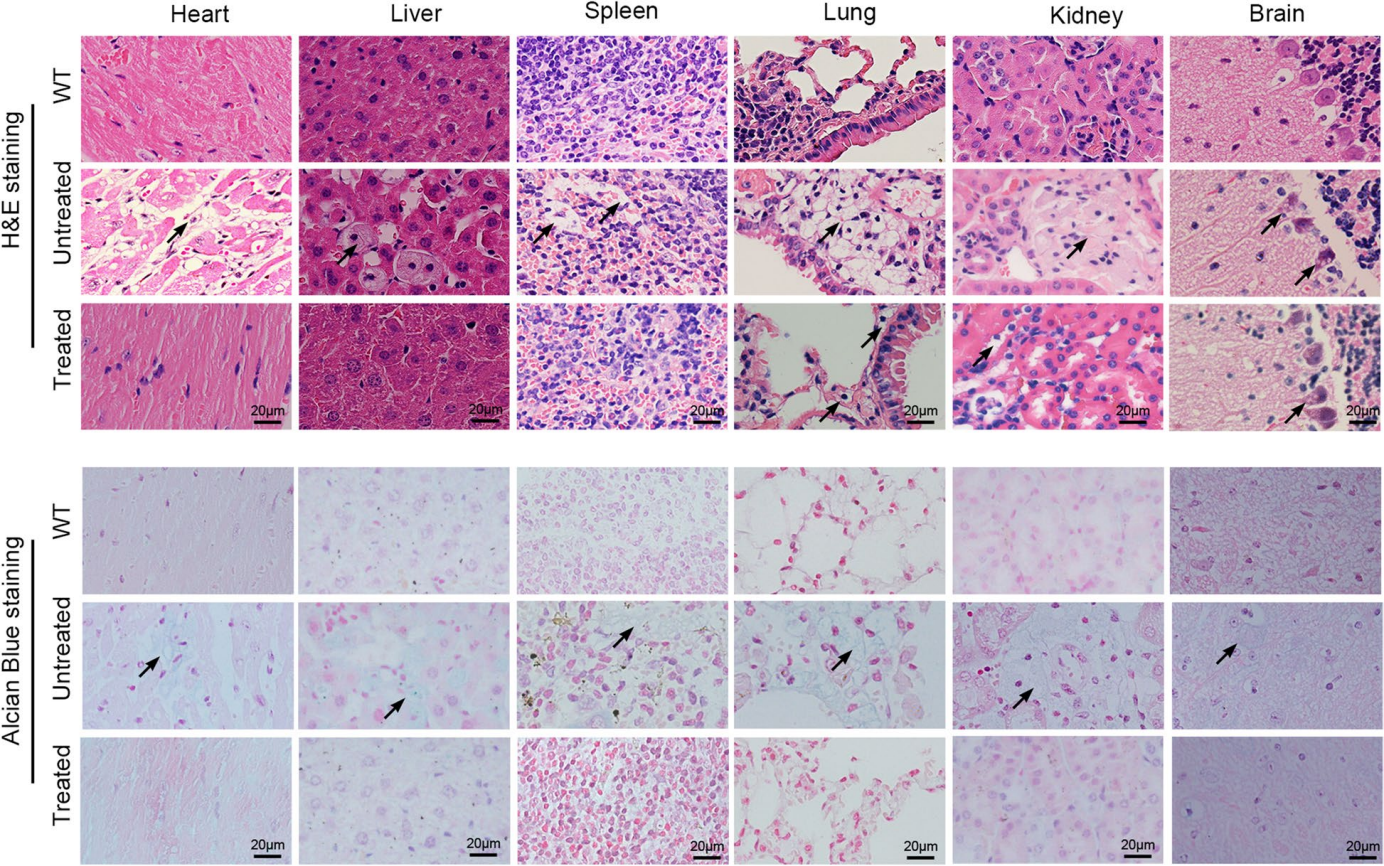
In vivo base editing corrects histological abnormalities in MPS IH mice. (Top) Histological analysis of the heart, liver, spleen, lung, kidney and brain tissues at 32 weeks post-injection by hematoxylin and eosin stain. Scale bar, 20 μm. Black arrows indicate foamy macrophages in the tissue due to GAGs accumulation. (Bottom) The tissues were stained with Alcian blue to detect GAGs. Scale bar, 20 μm. Black arrows indicate the GAGs storage in the tissues

The accumulation of GAGs in tissues leads to the formation of characteristic microscopic lysosomal vacuoles [34]. We performed a histological analysis of a subset of tissues (heart, liver, spleen, lung, kidney, and brain). In the hematoxylin and eosin (H&E) staining results, significant reduction of vacuolar cells was detected in the heart and liver tissues of the treated mice, and improvement in vacuolation of Purkinje cells was also observed (Fig. 5). A partial reduction in vacuolar cells was also observed in spleen, lung and kidney tissues (Fig. 5). To evaluate the correction of storage pathology in the treated mice, Alcian blue staining for GAGs was performed on tissue sections. As expected, GAGs storage was significantly higher in the tissues of untreated MPS IH mice than in those of WT mice. In accordance with the reduced pathological vacuolization, variably decreased Alcian blue staining of GAGs was detected in the heart, liver, spleen, lung and kidney tissues of treated mice (Fig. 5). We also observed a slight decrease in accumulated GAGs in the brains of treated MPS IH mice. In addition, histochemical analysis also showed there is no signs of inflammation such as lymphocyte or macrophage aggregation in tissues of the treated mice.

### In vivo base editing prevents neurobehavioral deficits in MPS IH mice

**Fig. 6:**
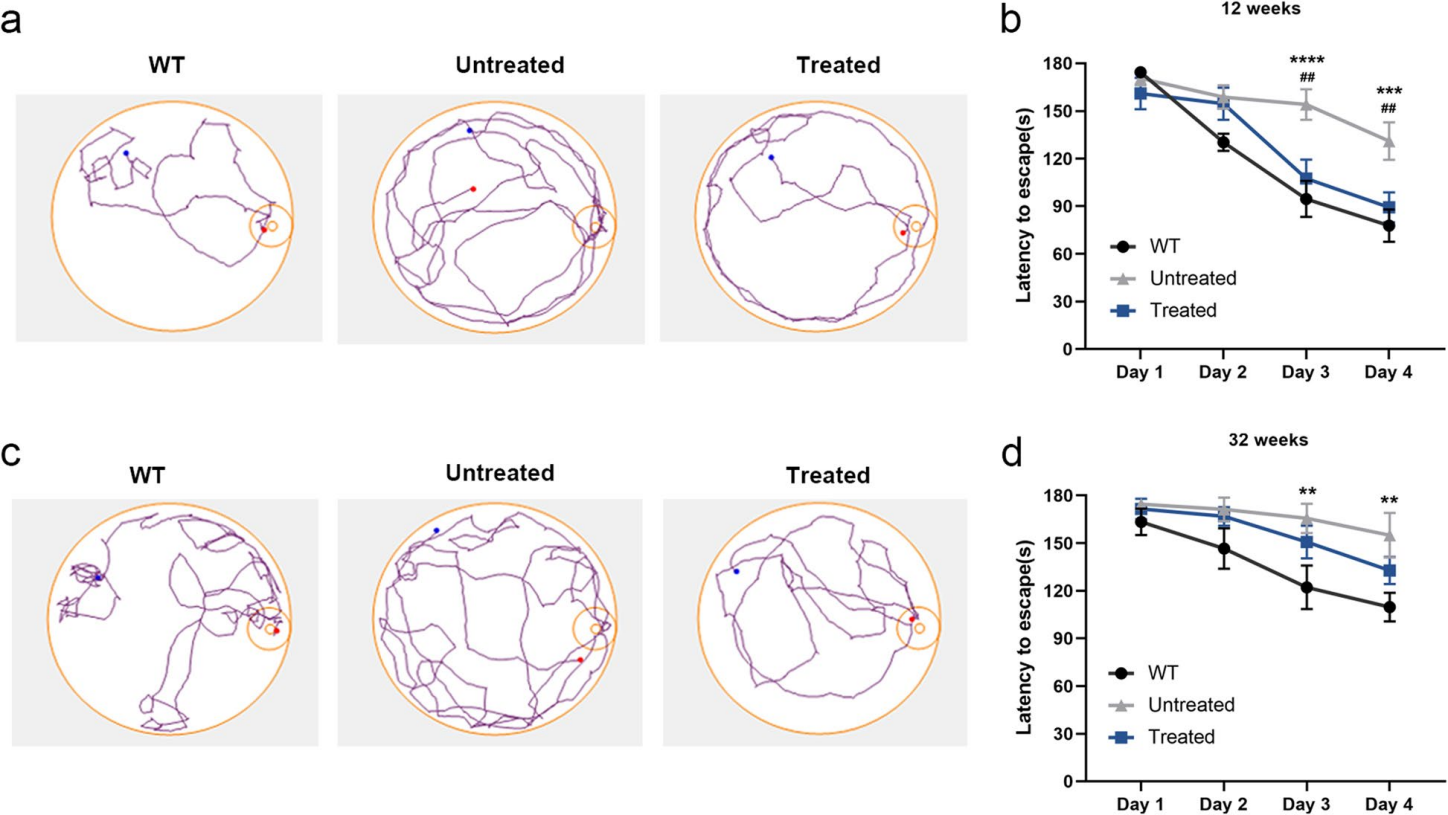
In vivo base editing prevents neurobehavioral deficit in MPS IH mice. Performance in the DMP dry maze is the time to escape from the maze. **a** Representative escape traces of 12-week-old mice in each group in the DMP dry maze. The blue dots indicate the original position of the mouse, and the red dots indicate the final position. **b** Quantitative analysis of average escape time after training in 12-week-old mice. Data were shown as mean ± SEM at each time point. WT mice (*n* = 6), untreated MPS IH mice (*n* = 6) and treated MPS IH mice (*n* = 5). **c** Representative escape traces of 32-week-old mice in each group in the DMP dry maze. **d** Quantitative analysis of average escape time after training in 32-week-old mice. Data were shown as mean ± SEM at each time point. WT mice (*n* = 7), untreated MPS IH mice (*n* = 7) and treated MPS IH mice (*n* = 7). (**b**, **d**) The WT mice were compared with the untreated MPS IH mice, **p < 0.01, ***p < 0.001, ****p < 0.0001. The treated MPS IH mice were compared with the untreated MPS IH mice, ##*p* < 0.01, one-way ANOVA analysis with Tukey’s post-hoc test

To detect whether base editors delivered via AAV9 provided any cognitive benefit to newborn MPS IH mice, we performed a delayed-matching-to-place (DMP) dry maze test 12 weeks and 32 weeks after injection. The DMP dry maze is a behavioral test that evaluates the learning and memory abilities of mice by measuring the time it takes for the mice to find an escape route on a high platform [33, 35]. The behavioral test results showed that there was no significant difference in the average speed of the mice in each group on the high platform (Supplemental Fig. 5). In the 12-week’ DMP test, the average escape latency of WT mice with normal cognitive functions was reduced from 177 s to 89 s after 4 days of testing and training. In contrast, untreated MPS IH mice showed a slow reduction in average escape latency from 170 s to 138 s, indicating cognitive deficits. Surprisingly, the escape of the treated mice was significantly faster on day 3 of the test, with no significant difference compared with the WT mice (Fig. 6a and b). Previous studies have reported that older mice spend more escape time than younger mice in the Barnes test because the mice’s spatial learning and memory abilities decline with age [36, 37]. In the 32- week’ DMP test, although the mice in each group spent more time to escape on the platform than 12-week-old mice, it was observed that the escape time of treated MPS IH mice (133 s) was less than that of age-matched untreated MPS IH mice (155 s) after training, and there was no significant difference when compared with WT mice (109 s) (Fig. 6c and d). These data suggested that in vivo base editing can partially prevent cognitive deficits in newborn MPS IH mice.

## Discussion

There is no or limited treatment options for rare disease patients around the world, most of whom are suffering from monogenic diseases caused by single-nucleotide variants (SNVs) [38]. Base editing has the potential to correct SNVs and can provide efficient and safe one-time treatment for many rare diseases [20]. Herein, we demonstrated that AAV9-mediated split-intein ABE could effectively correct pathogenic mutations in newborn MPS IH mice. We observed sustained serum IDUA activity and decreased tissue GAGs storage in MPS IH mice treated at neonatal stage. Moreover, the neurobehavioral deficits were partially prevented.

MPS IH is a multi-system disease involving the cardiovascular, skeletal, gastrointestinal, and nervous systems [39]. ERT is the most extensively used treatment in the attenuated forms of MPS I, but is not recommended for the severe Hurler phenotype because the enzyme cannot cross the blood-brain barrier to influence the central nervous manifestations and cannot completely correct heart valvular or bone disease [40, 41]. Despite early HSCT treatment may be able to prevent progressive neurocognitive impairment, the transplanted patients may still have a serious disease burden [42]. Therefore, it is necessary to find a safer and more effective method to treat MPS IH disease. An important feature of mucopolysaccharidoses (MPSs) is its relatively low therapeutic threshold, which is extremely beneficial for the development of gene therapy/gene-editing therapies for these diseases [43, 44]. To effectively treat central nervous manifestations of MPS IH and prevent anti-transgenic immune response, Hinderer et al. performed systemic transgenic treatment of neonates before intrathecal administration, which effectively treated brain storage lesions [13]. Vector dilution is a major problem in AAV gene therapy, which may lead to a gradual decline of therapeutic effect as the children grow [45]. In contrast, AAV-mediated base editors could irreversibly correct the pathogenic genes and have a sustained therapeutic effect.

In this study, we observed a high corrective efficiency in heart and liver tissues and improved disease outcomes. Additionally, we found that although the efficiency of genomic DNA correction in other tissues was low, the storage of GAGs was also reduced. One possible explanation is that MPS IH is a disease with a relatively low threshold for treatment [44]. The second possibility is that the IDUA enzyme expressed and secreted in the heart and liver tissues is transmitted through the blood to other tissues, thereby reducing the GAGs storage in these tissues. Surprisingly, the partial prevention of neurobehavioral deficits was detected in the treated MPS IH mice. We found a low vector copy numbers in the brain tissue, with a correction efficiency of about 0.34 ± 0.12%. Approximately 2.47% of the wild-type level of IDUA activity in the brain was observed in the treated mice. It is worth noting that only 0.5% of wild-type activity is required to prevent neurological complications of MPS IH [15]. A previous study has reported that the therapeutic effect of MPS IH brain treatment in neonatal mice is significantly better than that in adult mice, one of the important factors is likely the blood-brain barrier at different developmental stages [46]. The blood-brain barrier is known to be incomplete at birth, and mice and humans develop full barrier function within the first few weeks of life [47, 48]. Thus, the AAV9 vectors and IDUA may be more easily transferred from the bloodstream into the brain tissue in neonatal mice. MPS IH is a progressive multisystem disease. The accumulation of GAG and related pathological abnormalities have not occurred in the neonatal period. Base editing in the neonatal period is also “preventative”, which can slow down the accumulation rate of GAG, so as to continuously improve the late refractory phenotypes of MPS IH mice such as cardiac dysfunction, skeletal abnormality and neurobehavioral deficits.

In recent years, studies have reported that genome editing-mediated gene therapy could effectively repair the peripheral tissues and brain tissues of MPS I/MPS II mice [16, 49]. However, the production of high frequency indels limits its clinical application. Previous studies showed that base editors did not randomly induce untargeted base conversion throughout the genome, but might cause unexpected editing in the regions where the sgRNA/base editor complex binds to DNA due to sequence homology [50–52]. In this study, although A10 bystander editing was observed, it was a synonymous mutation that did not affect IDUA expression. Furthermore, we estimated the top 10 potential off-target sites identified by a computer algorithm. NGS revealed that indels were less than 0.2% in highly edited liver tissues, suggesting that our base editing strategy is safer in MPS IH treatment. A recent study of intrauterine base editing in the treatment of MPS IH mice has been reported, further confirming the effectiveness of base editing in MPS IH [53]. For progressive diseases such as MPS IH, early treatment is more helpful to improve disease outcomes. Many countries have introduced screening for neonatal lysosomal storage diseases. However, this screening is complicated by the wide clinical variability of these diseases and the fact that many people who are tested for enzyme deficiency will exhibit symptoms late or never in their lifetime [54]. In addition, the operation of intrauterine injection therapy is difficult and risky, and requires very professional experts and equipment. In our research, the therapeutic effect on newborn mice was significant and the operation was simple. Moreover, the base editing strategy can be further verified in the humanized animal model of *Idua* hotspot mutation, which has the potential for clinical translation.

In conclusion, our results suggest that AAV-mediated base editor delivery can effectively correct storage damage in multiple tissues of the genetic metabolic disease MPS IH and prevent neurobehavioral deficits. Currently, there are many optimized base editor variants that are not only more efficient for editing but are no longer actually restricted by the requirement of PAM for sequence recognition [55, 56]. Furthermore, Glycosylase base editors and CGBEs were developed to enable the transmutation of C to A and C to G [57–59]. We believe that base editing will become a favorable treatment for more genetic diseases caused by pathogenic mutations in the future.

## Material and methods

### Plasmid construction and cell transfection

VRQR-ABEmax (#119811), xCas9(3.7)-ABE(7.10) (#108382), NG-ABEmax (#124163), ABEmax(7.10)-SpG (#140002), NG-ABE8e (#138491) and pSPgRNA (#47108) plasmids were purchased from Addgene (Watertown, MA). To generate the ABE8e-SpG plasmid, ABE8e was digested by *NotI* and *EcoRV* restriction enzyme and subcloned into ABEmax(7.10)-SpG plasmid backbone by In-Fusion cloning (Takara Bio, Mountain View, CA). The HEK293-*Idua* mutant cell lines were generated by stably integrating *Idua*-W392X sequence into the AAVS1 locus. CRISPR/Cas9 plasmid used to generate the HEK293-*Idua* mutant cell lines was constructed using pX330 (Plasmid #42230) (Supplemental Table 3). sgRNA-A5 and sgRNA-A6 targeting the G → A W392X mutation site on exon 9 of *Idua* gene in the mouse genome were designed by online webtool (https://benchling.com). All sgRNAs constructed were generated by T4 ligation of annealed oligos into *BbsI* digested pSPgRNA plasmid. Next, six adenine base editors (VRQR-ABEmax, xCas9(3.7)-ABE (× 7.10), NG-ABEmax, ABEmax(7.10)-SpG, NG-ABE8e and ABE8e-SpG) were co-transfected with sgRNA-A5 or sgRNA-A6 into HEK293-*Idua* mutant cell lines, respectively. Genomic DNA was extracted 72 h after transfection and Sanger sequencing was performed to screen the most effective base editors. The sgRNA-A6 was selected to further engineering of the split-intein dual-AAV system (referred to as N-ABE8e.SpG and C-ABE8e.SpG.sgRNA-A6). Both N-ABE8e.SpG and C-ABE8e.SpG.sgRNA-A6 vectors used CBh promoter and were generated by In-Fusion cloning of PCR-amplified inserting into restriction enzyme-digested backbones. The coding sequences of split-intein ABE are shown in Supplemental Sequences. All constructed plasmids were verified by sequencing.

### AAV vector production

AAV9.C-ABE8e-SpG and AAV9.N-ABE8e-SpG were obtained by packaging N-ABE8e.SpG and C-ABE8e.SpG.sgRNA-A6 into an AAV9 vectors (Supplemental Sequences). All AAV9 vectors were produced by triple plasmid transfection of HEK293 cells (ATCC, Manassas, VA) as previously described [60]. The genome titer (genome copies [GCs] per milliliter, GC/ml) of AAV9 vector was determined by quantitative PCR (qPCR) using forward primer 5′-GCCAGCCATCTGTTGT-3′, reverse primer 5′-GGAGTGGCACCTTCCA-3′, and probe 5′-Fam- TCCCCCGTGCCTTCCTTGACC-Tamra-3′ [61]. All vectors used in this study passed the endotoxin assay using the QCL-1000 Chromogenic LAL test kit (Cambrex Bio Science).

### Western blot analysis

Western blot analyses were performed on cell lysates. SpCas9 protein was detected by Mouse anti-CRISPR-Cas9 antibody (1:1000 dilution, Abcam, Cat# 191468). Mouse anti-GAPDH antibody (1:10000 dilution, ABclonal, Cat# AC002) was used to detect GAPDH. Blots were imaged and analyzed by iBrightTM CL1000 imaging systems (Thermo FisherScientific, Invitrogen™).

### Animal studies

MPS IH mice (*Idua*-W392X, Stock No: 017681) were purchased from Jackson Laboratory (Bar Harbor, Maine). The background of the WT mice used in this study were C57BL/6 J. Mating cages were monitored daily for births. Newborn (postnatal day 2, p2) pups received a temporal vein injection of the mixture of AAV9.C-SpG8e-SpG and AAV9.N-SpG8e-SpG at 1:1 (3 × 10^11^GC /mouse for each vector) in a volume of 50 μl, as described [62]. WT mice, MPS I heterozygous (Het), and untreated MPS I mice (*Idua*-W392X) served as controls. Mice were genotyped at weaning to confirm genotype. Serum samples for IDUA enzyme activity assays were obtained by retro-orbital bleeding 4 weeks post vector treatment and every 1 to 2 weeks thereafter. Urine samples were collected by gently applying pressure to the urinary bladder at the time of necropsy. The mice were killed at 12 and 32 weeks of age and tissues were collected for various analysis.

### IDUA enzyme activity assay

Tissue and serum samples were immediately frozen on dry ice and stored at − 80 °C until analysis. Serum was used directly in IDUA enzyme activity assays. Tissue samples were homogenized in lysis buffer (0.9% NaCl, 0.2% Triton-X100, pH 3.5), freeze-thawed and clarified by centrifugation. Protein concentrations were determined by BCA protein assay (Thermo Scientific, Waltham, MA). IDUA enzyme activity was determined in a fluorometric assay using the synthetic substrate 4MU-iduronide (Glycosynth, Warrington, England) as previously described [14]. Units are given as nmol 4MU liberated per hour per mg of protein (tissues) or per ml of serum.

### Tissue GAGs assay

Tissue samples were consistent with IDUA enzyme assays. Tissue GAGs were determined using the Blyscan Glycosaminoglycan Assay Kit (Biocolor, Carrickfergus, UK), according to the manufacturer’s instructions.

### AAV9 biodistribution

DNA was extracted from tissues and total vector genomes quantified by Taqman qPCR as previously described [62].

### On-target and off-target analysis

To evaluate the on-target editing efficiency of various tissues, the tissue genomic DNA was extracted and then amplified by nest PCR to obtain the sequence fragment containing the W392X mutation, which was then analyzed by NGS. Furthermore, the top 10 potential off-target sites for sgRNA6 were identified by the algorithm described in www.benchling.com (Supplemental Table 1). These off-target sites were amplified by nest PCR in the liver tissue genomic DNA and deep sequenced with NGS. Libraries were made from the second PCR products and sequenced on Illumina Miseq (2 × 300 bp paired end, Personal Biotechnology Co., Ltd., Shanghai, China). Data were processed according to standard Illumina sequencing analysis procedures. Processed reads were mapped to the expected PCR amplicons as reference sequences using custom scripts. Reads that did not map to reference were discarded. Indels were determined by comparison of reads against reference using custom scripts.

### Echocardiography

Thirty-two weeks after treatment, high-frequency echocardiography was performed to assess the cardiac function by using a Vevo®3100 system ((Fujifilm VisualSonics, Ontario, Canada). Mice were anesthetized with isoflurane and placed on the warming platform in the supine position with a heart rate between 400 and 500 beats per minute. M-mode images were recorded in the short-axis view of the LV to assess LV function and dimensions and the aortic arch was measured with a modified suprasternal view. The LV fractional shortening, ejection fraction, heart rate, LV end-systolic diameter and LV end-diastolic diameter, etc. were analyzed with Vevo LAB LV analysis tool .

### Micro-CT

To detect whether base editing could improve skeletal dysplasia in MPS IH mice, the micro-CT (Quantum GX, PerkinElmer, Waltham, MA) was used to scan the zygomata and femora of the mice. Mice were anesthetized with isoflurane and placed in the CT chamber for scanning. Images were analyzed using the ImageJ program.

### H&E staining

Tissues were fixed in paraformaldehyde for 24 h, dehydrated through an ethanol series and xylene, and then embedded in paraffin. H&E staining was performed on 6 μm sections from paraffin-embedded tissues according to standard protocols.

### GAGs histochemistry

Tissue samples were prepared as H&E staining. Deparaffinized 6 μm sections were stained in 1% Alcian Blue (Sigma, #MKCM1030) for 15 minutes, rinsed in water for 2-3 minutes, and counterstained with Nuclear Fast Red (Sigma, #N8002).

### DMP dry maze assay

To detect whether base editors delivered via AAV9 provided any cognitive benefit to MPS IH mice, we performed a DMP dry maze test 12 weeks and 32 weeks after injection. DMP dry maze test was a variant of DMP water maze [63]. The DMP dry maze was a circular platform (Diameter = 122 cm, thickness = 1.2 cm) with 40 holes. An escape pipe was secured under one of the holes to allow the mice to escape the platform. The location of the escape hole changed every day. Visual cues were attached to each of the four walls for the mouse to use in spatial navigation. To begin the experiment, mice were placed on the edge of a platform some distance from the escape hole, and an opaque funnel covered the mouse. After a delay of about 30 sec, turning on the tone noise (2 KHz, 85 dB) and immediately removing the transfer box to expose mice in a bright light (1200 Lux). In response to these aversive conditions, the mice would spontaneously seek out and burrow into the escape hole. Mice were assessed during four trials per day on four consecutive days, with a maximal escape time limited to 3 min. Data were collected and analyzed using the ANY-Maze program.

### Statistics

Graphpad Prism9 was used to perform all statistical tests. Data are presented as mean ± SD in Figs. 1, 2, 3 and 4 and Supplemental Figs. 2A and 3). Data are presented as mean ± SEM in Fig. 6B, C and Supplemental Fig. 5. One-way ANOVA with Tukey’s post-hoc test was used in Figs. 1H, 2B-D, 3, 4C-E and Supplemental Figs. 3 and 4C-E. Two-way ANOVA with Tukey’s post-hoc test in Fig. 6B, C and Supplemental Fig. 5. In all tests, *p* < 0.05 was considered significant.

## Supplementary Information

**Additional file 1: Fig. S1.** Construction of HEK293-Idua mutant cell line using CRISPR/Cas9. a Schematic diagram of HEK293-Idua mutant cell line construction. b Screening of sgRNA in the construction of mutant cell lines. In vitro validation of the editing effect of sgRNAs in the HEK293 cell line by transient transfection and SURVEYOR nuclease assays. Arrows denote SURVEYOR nuclease cleaved fragments of the AAVS1 PCR products. Asterisks indicate nonspecific bands. c Gel electrophoresis verified that the mutant sequence was successfully inserted into the DNA genome of HEK293 cells. The inserted mutant sequences are marked in red. d The successful construction of HEKK293-Idua mutant cell line was verified by Sanger sequencing. The shaded part is the mutation site. **Fig. S2.** In vitro validation of the split-intein base editor. a Sanger sequencing analysis of split-intein ABE8e-SpG correction efficiency in mutant cell lines. Transfection of full-length ABE8e-SpG serves as control (n = 3 biological replicates each). Mean ± SD are shown. b Western blot analysis of co-transfected split-intein ABE8e-SpG. The SpCas9 epitope is only detected at the N-terminal part of the base editor. **Fig. S3.** In vivo base editing enables biochemical corrections in treated MPS IH mice 12 weeks after injection. a Tissue IDUA activity was detected in various tissues 12 weeks after injection. b Tissue GAGs storage was detected in various tissues 12 weeks after injection. (a, b) WT mice (n = 6) and untreated MPS IH mice (n = 6) were included as control. Treated MPS IH mice (n = 5). Mean ± SD are shown. The treated MPS IH mice were compared with the untreated MPS IH mice, #*p* < 0.05, ##*p* < 0.01, ####*p* < 0.0001, one-way ANOVA analysis with Tukey’s post-hoc test. **Fig. S4.** Detection of skeletons of WT mice, untreated and treated MPS IH mice 12 weeks after injection. a Representative micro-CT images of 12-week-old mice showing zygomatic arches (white arrows). Scale bar, 2 mm. b Representative micro-CT image of a 12-week-old mouse showing the femur. The two white arrows in the same image indicate the width of the femur. Scale bar, 1 mm. c-e Quantification of zygomatic arch width, femur width and femur length. Mean ± SD are shown. WT mice (n = 6), untreated MPS IH mice (n = 6) and treated MPS IH mice (n = 5). The WT mice and treated MPS I mice were compared with the untreated MPS I mice. There were no significant differences between the groups. One-way ANOVA analysis with Tukey’s post-hoc test. **Fig. S5.** Average running speed for all groups on four-day behavioral testing 12 weeks and 32 weeks after injection. To confirm that the deficits displayed by the MPS IH mice were not due to motor ability deficits caused by physical illness, statistics on the average running speed of all groups were performed. a Quantitative analysis of running speed of each group of mice 12 weeks after injection. Data were shown as mean ± SEM at each time point. WT mice (n = 6), untreated MPS IH mice (n = 6) and treated MPS IH mice (n = 5). b Quantitative analysis of running speed of each group of mice 32 weeks after injection. WT mice (n = 7), untreated MPS IH mice (n = 7) and treated MPS IH mice (n = 7). Data were shown as mean. The WT mice and treated MPS IH mice were compared with the untreated MPS IH mice. There were no significant differences between the groups. One-way ANOVA analysis with Tukey’s post-hoc test. **Supplemental Table 1.** Primers and sequences for construction of HEK293-Idua mutant cell lines. **Supplemental Table 2.** Off-target analysis. Potential off-target sequences for sgRNA-A6 identified and scored by Benchling’s off-target analysis. **Supplemental Table 3.** PCR primer sequences for detecting potential on-target and off-target effects by NGS assay.

## Data Availability

Data supporting the studies presented in this manuscript can be found in the main text or the supplemental information. Additional information may be requested from the corresponding authors as appropriate.
