# Supplementary material for: In vivo adenine base editing corrects newborn murine model of Hurler syndrome
Source: Mol Biomed. 2023 Feb 23;4:6. doi: 10.1186/s43556-023-00120-8 (PMC9947215; doi:10.1186/s43556-023-00120-8)
Supplement: Supplementary file 1 — Additional file 1: Fig. S1. Construction of HEK293-Idua mutant cell line using CRISPR/Cas9. a Schematic diagram of HEK293-Idua mutant cell line construction. b Screening of sgRNA in the construction of mutant cell lines. In vitro validation of the editing effect of sgRNAs in the HEK293 cell line by transient transfection and SURVEYOR nuclease assays. Arrows denote SURVEYOR nuclease cleaved fragments of the AAVS1 PCR products. Asterisks indicate nonspecific bands. c Gel electrophoresis verified that the mutant sequence was successfully inserted into the DNA genome of HEK293 cells. The inserted mutant sequences are marked in red. d The successful construction of HEKK293-Idua mutant cell line was verified by Sanger sequencing. The shaded part is the mutation site. Fig. S2. In vitro validation of the split-intein base editor. a Sanger sequencing analysis of split-intein ABE8e-SpG correction efficiency in mutant cell lines. Transfection of full-length ABE8e-SpG serves as control (n = 3 biological replicates each). Mean ± SD are shown. b Western blot analysis of co-transfected split-intein ABE8e-SpG. The SpCas9 epitope is only detected at the N-terminal part of the base editor. Fig. S3. In vivo base editing enables biochemical corrections in treated MPS IH mice 12 weeks after injection. a Tissue IDUA activity was detected in various tissues 12 weeks after injection. b Tissue GAGs storage was detected in various tissues 12 weeks after injection. (a, b) WT mice (n = 6) and untreated MPS IH mice (n = 6) were included as control. Treated MPS IH mice (n = 5). Mean ± SD are shown. The treated MPS IH mice were compared with the untreated MPS IH mice, #p < 0.05, ##p < 0.01, ####p < 0.0001, one-way ANOVA analysis with Tukey’s post-hoc test. Fig. S4. Detection of skeletons of WT mice, untreated and treated MPS IH mice 12 weeks after injection. a Representative micro-CT images of 12-week-old mice showing zygomatic arches (white arrows). Scale bar, 2 mm. b Representative micro-CT ima [file 43556_2023_120_MOESM1_ESM.pdf]

In vivo adenine base editing corrects newborn murine model of Hurler syndrome

Jing Su[1#], Xiu Jin[1#], Kaiqin She[1,2#], Yi Liu[1], Li Song[1], Qinyu Zhao[1], Jianlu Xiao[1], Ruiting Li[1], Hongxin Deng[1], Fang Lu[2], Yang Yang[1]*

[1]State Key Laboratory of Biotherapy and Cancer Center, West China Hospital, Sichuan University and Collaborative Innovation Center, Chengdu, Sichuan, China

[2]Department of Ophthalmology, West China Hospital, Sichuan University, Chengdu, Sichuan, China

[#]These authors contributed equally: Jing Su, Xiu Jin, Kaiqin She

*Corresponding authors: Yang Yang

State Key Laboratory of Biotherapy and Cancer Center, West China Hospital, Sichuan University and Collaborative Innovation Center, Chengdu 610041, China,

Postal address: Ke-yuan Road 4, No. 1, Gao-peng Street, Chengdu, Sichuan, 610041, China

E-mail: yang2012@scu.edu.cn

Tel: + 86 028 85164063

Supplemental Figure 1

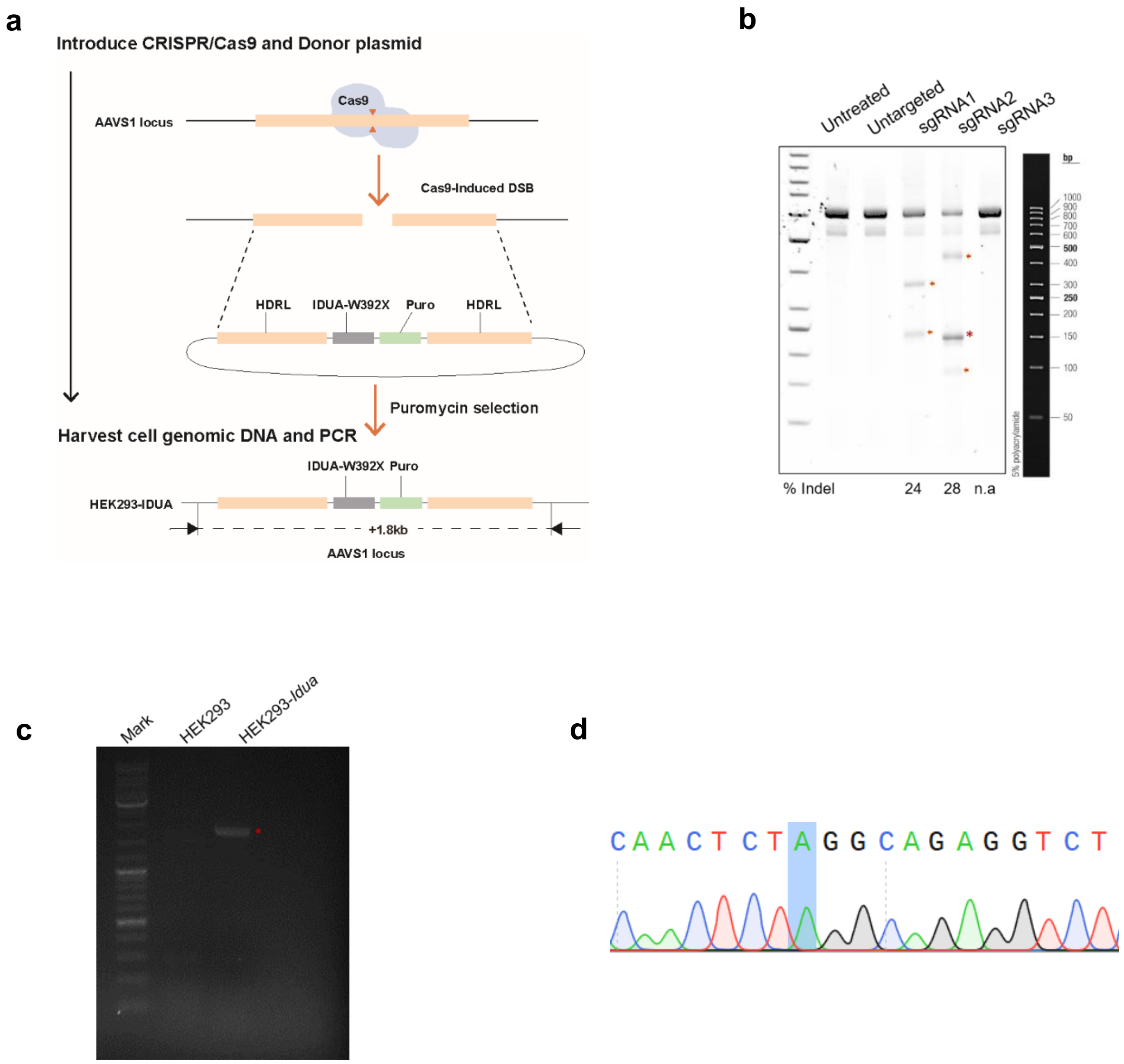

Fig.S1. Construction of HEK293-*Idua* mutant cell line using CRISPR/Cas9. a Schematic diagram of HEK293-*Idua* mutant cell line construction. **b** Screening of sgRNA in the construction of mutant cell lines. *In vitro* validation of the editing effect of sgRNAs in the HEK293 cell line by transient transfection and SURVEYOR nuclease assays. Arrows denote SURVEYOR nuclease cleaved fragments of the AAVS1 PCR products. Asterisks indicate nonspecific bands. **c** Gel electrophoresis verified that the mutant sequence was successfully inserted into the DNA genome of HEK293 cells. The inserted mutant sequences are marked in red. **d** The successful construction of HEKK293-*Idua* mutant cell line was verified by Sanger sequencing. The shaded part is the mutation site.

Supplemental Figure 2

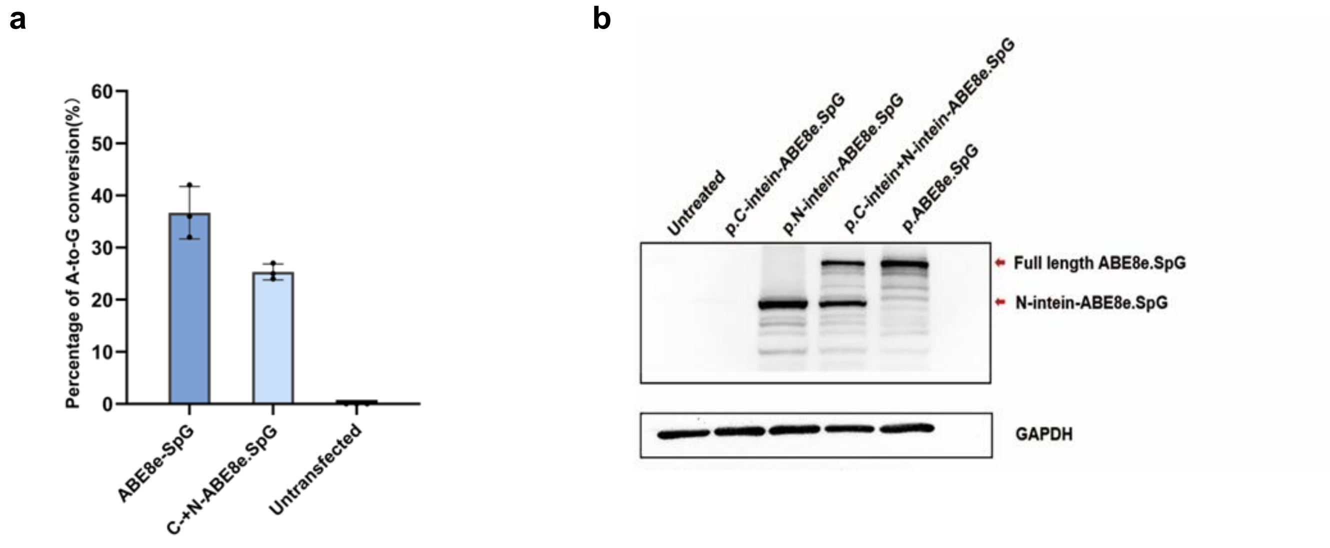

Fig.S2. *In vitro* validation of the split-intein base editor. a Sanger sequencing analysis of split-intein ABE8e-SpG correction efficiency in mutant cell lines. Transfection of full-length ABE8e-SpG serves as control (n=3 biological replicates each). Mean ± SD are shown. **b** Western blot analysis of co-transfected split-intein ABE8e-SpG. The SpCas9 epitope is only detected at the N-terminal part of the base editor.

Supplemental Figure 3

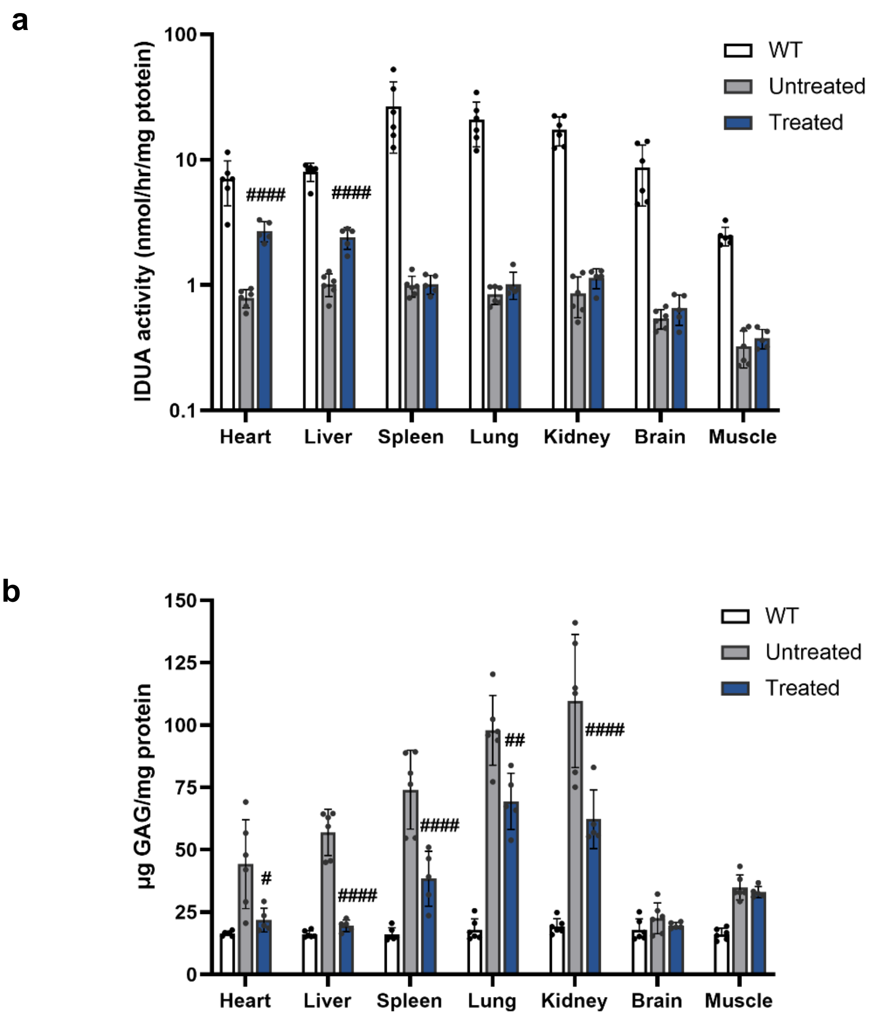

Fig.S3. *In vivo* base editing enables biochemical corrections in treated MPS IH mice 12 weeks after injection. a Tissue IDUA activity was detected in various tissues 12 weeks after injection. **b** Tissue GAGs storage was detected in various tissues 12 weeks after injection. (**a, b**) WT mice (n=6) and untreated MPS IH mice (n=6) were included as control. Treated MPS IH mice (n=5). Mean ± SD are shown. The treated MPS IH mice were compared with the untreated MPS IH mice, #p<0.05, ##p<0.01, ####p<0.0001, one-way ANOVA analysis with Tukey's post-hoc test.

Supplemental Figure 4

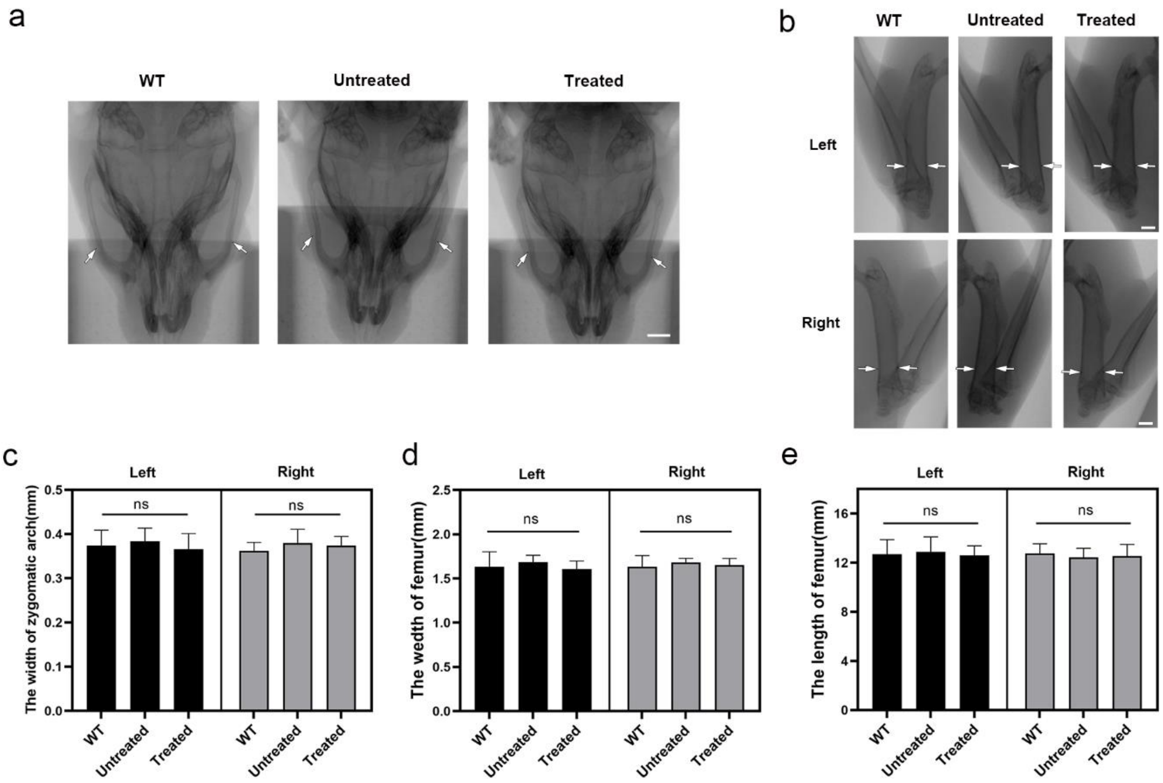

Fig.S4. Detection of skeletons of WT mice, untreated and treated MPS IH mice 12 weeks after injection. a Representative micro-CT images of 12-week-old mice showing zygomatic arches (white arrows). Scale bar, 2 mm. **b** Representative micro-CT image of a 12-week-old mouse showing the femur. The two white arrows in the same image indicate the width of the femur. Scale bar, 1 mm. **c-e** Quantification of zygomatic arch width, femur width and femur length. Mean ± SD are shown. WT mice (n=6) , untreated MPS IH mice (n=6) and treated MPS IH mice (n=5). The WT mice and treated MPS I mice were compared with the untreated MPS I mice. There were no significant differences between the groups. One-way ANOVA analysis with Tukey's post-hoc test.

Supplemental Figure 5

a

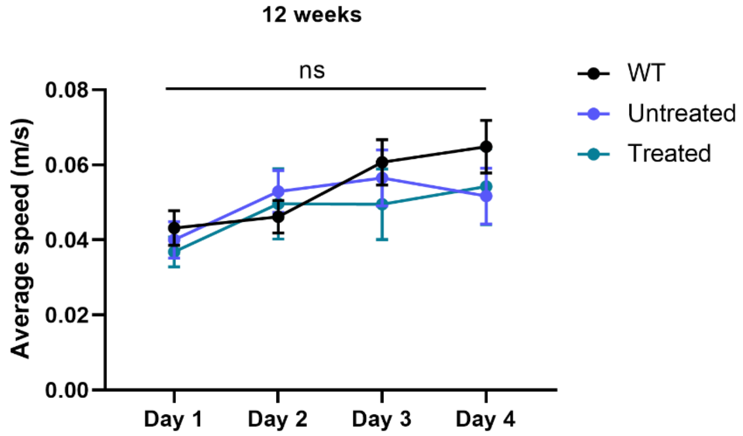

b

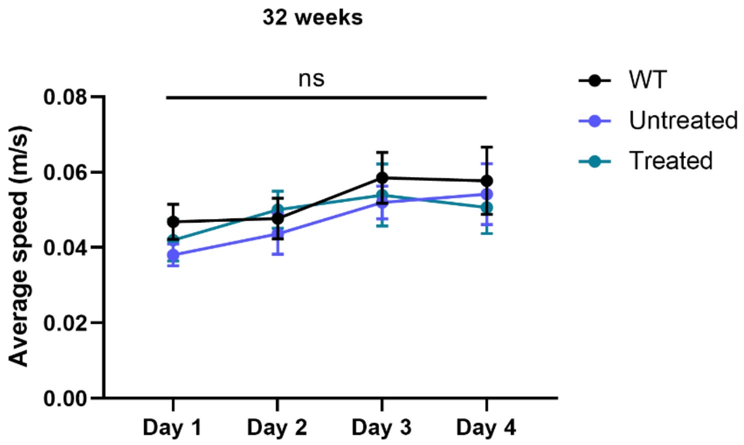

Fig.S5. Average running speed for all groups on four-day behavioral testing 12 weeks and 32 weeks after injection. To confirm that the deficits displayed by the MPS IH mice were not due to motor ability deficits caused by physical illness, statistics on the average running speed of all groups were performed. **a** Quantitative analysis of running speed of each group of mice 12 weeks after injection. Data were shown as mean ±SEM at each time point. WT mice (n=6), untreated MPS IH mice (n=6) and treated MPS IH mice (n=5). **b** Quantitative analysis of running speed of each group of mice 32 weeks after injection. WT mice (n=7), untreated MPS IH mice (n=7) and treated MPS IH mice (n=7). Data were shown as mean. The WT mice and treated MPS IH mice were compared with the untreated MPS IH mice. There were no significant differences between the groups. One-way ANOVA analysis with Tukey's post-hoc test.

Supplemental Table 1. Primers and sequences for construction of HEK293-*Idua* mutant cell lines.

**Name**	**Sequence**	**Note**
sgRNA1	GGGACCACCTTATATTCCCA (PAM: GGG)	Three sgRNA sequences for construction of HEK293-*Idua* mutant cell lines
sgRNA2	GAGATGGCTCCAGGAAATGG (PAM: GGG)	
sgRNA3	TAAGGAATCTGCCTAACAGG (PAM: AGG)	
AAVS1_T1 Fwd	CACCGGGACCACCTTATATTCCCA	CRISPR/Cas9 plasmid-1 construction using pX330
AAVS1_T1 Rev	AAACTGGGAATATAAGGTGGTCCC	
AAVS1_T2Fwd	CACCGAGATGGCTCCAGGAAATGG	CRISPR/Cas9 plasmid-2 construction using pX330
AAVS1_T2 Rev	AAACCCATTTCCTGGAGCCATCTC	
AAVS1_T3 Fwd	CACCGTAAGGAATCTGCCTAACAGG	CRISPR/Cas9 plasmid-3 construction using pX330
AAVS1_T3 Rev	AAACCCTGTTAGGCAGATTCCTTAC	
AAVS1_ P1Fwd	TCCTGAGTCCGGACCACTTT	The primers of surveyor assay for validation of sgRNA
AAVS1_ P1Rev	GCTTCTTGGCCACGTAACCT	
AAVS1_ PointMF	TTTGCCTGGACACCCCGTTC	
AAVS1_ PointMR	GCGTCAGAGCAGCTCAGGTT	
MPS1_Fwd	CCTGGCACATCCTGTATTGA	PCR Primers for HEK293-*Idua* mutant cell lines
MPS1_Rev	CCTCTACAAATGTGGTATGGC	

Supplemental Table 2. Off-target analysis. Potential off-target sequences for sgRNA-A6 identified and scored by Benchling's off-target analysis.

ID	Sequence	PAM	Score	Chromosome	Strand	Position	Mismatches
**SgRNA-A6**	ACTCTAGGCAGAGGTCTCAA	AG	100.00	Chr5	1	108681407	0
**OT1**	TGGCTAGGCAGAGGTCTCAA	TG	2.32	Chr11	-1	72424740	3
**OT2**	AATGTATGCAGAGGTCTCAA	GG	1.77	Chr6	-1	76674529	3
**OT3**	TCTCTAGAAAGAGGTCTCAA	AG	1.75	Chr8	-1	34402855	3
**OT4**	ACTTTATGCTGAGGTCTCAA	TG	1.68	Chr7	-1	64896671	3
**OT5**	ACTCCAGCAAGAGGTCTCAA	AG	1.53	chr12	-1	81903353	3
**OT6**	GTTCTAGACTGAGGTCTCAA	GG	1.50	Chr17	-1	8999275	4
**OT7**	AGTTCAGACAGAGGTCTCAA	AG	1.44	Chr9	-1	26721248	4
**OT8**	ATCCAAGGCTGAGGTCTCAA	TG	1.39	chr17	1	33748567	4
**OT9**	TTACAAGGCAGAGGTCTCAA	TG	1.36	chr11	-1	111283516	4
**OT10**	TTCCCAGGCAGAGGTCTCAA	AG	1.36	Chr8	1	69133495	4

Supplemental Table 3. PCR primer sequences for detecting potential on-target and off-target effects by NGS assay.

Primer Name	Sequence	Note
**On-target**		
Nest_P1Fwd	AGTTGCTGCGAAAGCCAGTA	Primers for on-target
Nest1_P1Rev	AGGGATACTGTGGTTGGGGT	
Nest2_P2FWD	GGTGGGAGCTAGATATTAGG	
Nest2_P2Rev	AGATGAGGACTGTGGTACTC	
**Off-target**		
OT1_P1Fwd	ACTGGAAGAAACAGCGGAAG	Primers for off-target1
OT1_P1Rev	GAGTGTGAGACCTCTAAGAG	
OT1_P2Fwd	AGCTGGGGAACAAGTGTTCA	
OT1_P2Rev	GCTGGATAACTACACCGTTC	
OT2_P1Fwd	CACTGGTTTCCAAGACTAAGG	Primers for off-target2
OT2_P1Rev	AGGTTAAGTGCAGAAAGGAG	
OT2_P2Fwd	ACCAATACAGTAGCCCTATG	
OT2_P2Rev	GCAGAAAGGAGATATTTCTCTGAG	
OT3_P1Fwd	CTCTAGCTCTCATGCACACA	Primers for off-target3
OT3_P1Rev	CCCTCAGTGGCAACCTATAA	
OT3_P2Fwd	TTGGAGACCTGTGACTAGGA	
OT3_P2Rev	TGGGTCACTTGAGTTCTTCC	
OT4_P1Fwd	CCGTTGTGAGTGTGCCATTT	Primers for off-target4
OT4_P1Rev	GATCTGTCCACTGATCCACT	
OT4_P2Fwd	GACCAAACAAGGACTTGTCA	
OT4_P2Rev	GCACACTTAGGTCTACCTTC	
OT5_P1Fwd	GGCAAGACAATCTTGCTCAC	Primers for off-target5
OT5_P1Rev	TACGTGGCAGTGAGGAATTG	
OT5_P2Fwd	GTGTTTCTGATGAAACTTCC	
OT5_P2Rev	AGGAGCAGATTCTCTCACAC	
OT6_P1Fwd	GAACTGAGAGGAGAGCGAAA	Primers for off-target6
OT6_P1Rev	CCAGAAGGCTCTCTGCATTT	
OT6_P2Fwd	GTAAGAGTGGGCCATGTGAA	
OT6_P2Rev	ATTCTGGCAGGTGCTGCATGTA	
OT7_P1Fwd	GATCACACAATGGGCACTCT	Primers for off-target7
OT7_P1Rev	GAGTGGTTGGAAGTTCAAGC	
OT7_P2Fwd	TTCTATCCTGTTGGGAGGCA	
OT7_P2Rev	ATAGGGTGTTGAGATTCTGG	
OT8_P1Fwd	GTTAGCTGCTTCTCCCTAGA	Primers for off-target8
OT8_P1Rev	ACCTGAGTCACTGTTCCATC	
OT8_P2Fwd	CTGTGCTCTGTATCTGCACA	
OT8_P2Rev	ACAGTTGCATCCTGGAGCTA	
OT9_P1Fwd	AGGCTGCTTGTTCCTTCCTT	Primers for off-target9

OT9_P1Rev	CAGTGTCAAGGCTCATAGTC	
OT9_P2Fwd	ACTGGGTCCTACACATAAAG	
OT9_P2Rev	TGGGAGCCAGACCCTCTTATTT	
OT10_P1Fwd	TCCTACTCTCTTAACTCCC	Primers for off-target10
OT10_P1Rev	TCTATTCAGTGTGCCCTGGT	
OT10_P2Fwd	CTTCCCCCATACCTGATTAT	
OT10_P2Rev	TGCCTCCAAGGAAAGACTCA	

Supplemental Sequences. Coding sequences of split-intein ABE

Coding sequence for N-ABE8e.SpG-Int^{N}

MKRTADGSEFESPKKKRKVSEVEFSHEYWMRHALTLAKRARDEREVPVGAVLVLNNRVIGEGWNRAIGL
HDPTAHAEIMALRQGGLVMQNYRLIDATLYVTFEPCVMCAGAMIHSRIGRVVFGVRNSKRGAAGSLMNV
LNYPGMNHRVEITEGILADECAALLCDFYRMPRQVFNAQKKAQSSINSGGSSGGSSGSETPGTSESATPES
SGGSSGGSDKKYSIGLAIGTNSVGWAVITDEYKVPSKKFKVLGNTDRHSIKKNLIGALLFDSGETAEATRL
KRTARRRYTRRKNRICYLQEIFSNEMAKVDDSFFHRLEESFLVEEDKKHERHPIFGNIVDEVAYHEKYPTIY
HLRKKLVDSTDKADLRLIYLALAHMIKFRGHFLIEGDLNPDNSDVDKLFIQLVQTYNQLFEENPINASGVD
AKAILSARLSKSRRLENLIAQLPGEKKNGLFGNLIALSLGLTPNFKSNFDLAEDAKLQLSKDTYDDDLDNL
LAQIGDQYADLFLAAKNLSDAILLSDILRVNTEITKAPLSASMIKRYDEHHQDLTLLKALVRQQLPEKYKEI
FFDQSKNGYAGYIDGGASQEEFYKFIKPILEKMDGTEELLVKLNREDLLRKQRTFDNGSIPHQIHLGELHAI
LRRQEDFYPFLKDNREKIEKILTFRIPYYVGPLARGNSRFAWMTRKSEETITPWNFEEVVDKGASAQSFIER
MTNFDKNLPNEKVLPKHSLLYEYFTVYNELTKVKYVTEGMRKPAFLSGEQKKAIVDLLFKTNRKVTVKQ
LKEDYFKKIECLSYETEILTVEYGLLPIGKIVEKRIECTVYSVDNNGNIYTQPVAQWHDRGEQEVFEYCLED
GSLIRATKDHKFMTVDGQMLPIDEIFERELDLMRVDNLPN

ATGAAACGGACAGCCGACGGAAGCGAGTTCGAGTCACCAAAGAAGAAGCGGAAAGTCTCTGAGGTG
GAGTTTTCCCACGAGTACTGGATGAGACATGCCCTGACCCTGGCCAAGAGGGCACGGGATGAGAGGG
AGGTGCCTGTGGGAGCCGTGCTGGTGCTGAACAATAGAGTGATCGGCGAGGGCTGGAACAGAGCCAT
CGGCCTGCACGACCCAACAGCCCATGCCGAAATTATGGCCCTGAGACAGGGCGGCCTGGTCATGCAG
AACTACAGACTGATTGACGCCACCCTGTACGTGACATTCGAGCCTTGCGTGATGTGCGCCGGCGCCAT
GATCCACTCTAGGATCGGCCGCGTGGTGTTTGGCGTGAGGAACTCAAAAAGAGGCGCCGCAGGCTCC
CTGATGAACGTGCTGAACTACCCCGGCATGAATCACCGCGTCGAAATTACCGAGGGAATCCTGGCAGA
TGAATGTGCCGCCCTGCTGTGCGATTTCTATCGGATGCCTAGACAGGTGTTCAATGCTCAGAAGAAGG
CCCAGAGCTCCATCAACTCCGGAGGATCTAGCGGAGGCTCCTCTGGCTCTGAGACACCTGGCACAAG
CGAGAGCGCAACACCTGAAAGCAGCGGGGGCAGCAGCGGGGGGTCAGACAAGAAGTACAGCATCGG
CCTGGCCATCGGCACCAACTCTGTGGGCTGGGCCGTGATCACCGACGAGTACAAGGTGCCCAGCAAG
AAATTCAAGGTGCTGGGCAACACCGACCGGCACAGCATCAAGAAGAACCTGATCGGAGCCCTGCTGT
TCGACAGCGGCGAAACAGCCGAGGCCACCCGGCTGAAGAGAACCGCCAGAAGAAGATACACCAGAC
GGAAGAACCGGATCTGCTATCTGCAAGAGATCTTCAGCAACGAGATGGCCAAGGTGGACGACAGCTT
CTTCCACAGACTGGAAGAGTCCTTCCTGGTGGAAGAGGATAAGAAGCACGAGCGGCACCCCATCTTC
GGCAACATCGTGGACGAGGTGGCCTACCACGAGAAGTACCCCACCATCTACCACCTGAGAAAGAAAC
TGGTGGACAGCACCGACAAGGCCGACCTGCGGCTGATCTATCTGGCCCTGGCCCACATGATCAAGTTC
CGGGGCCACTTCCTGATCGAGGGCGACCTGAACCCCGACAACAGCGACGTGGACAAGCTGTTCATCC
AGCTGGTGCAGACCTACAACCAGCTGTTCGAGGAAAACCCCATCAACGCCAGCGGCGTGGACGCCAA
GGCCATCCTGTCTGCCAGACTGAGCAAGAGCAGACGGCTGGAAAATCTGATCGCCCAGCTGCCCGGC
GAGAAGAAGAATGGCCTGTTCGGAAACCTGATTGCCCTGAGCCTGGGCCTGACCCCCAACTTCAAGA
GCAACTTCGACCTGGCCGAGGATGCCAAACTGCAGCTGAGCAAGGACACCTACGACGACGACCTGG
ACAACCTGCTGGCCCAGATCGGCGACCAGTACGCCGACCTGTTTCTGGCCGCCAAGAACCTGTCCGA
CGCCATCCTGCTGAGCGACATCCTGAGAGTGAACACCGAGATCACCAAGGCCCCCCTGAGCGCCTCT
ATGATCAAGAGATACGACGAGCACCACCAGGACCTGACCCTGCTGAAAGCTCTCGTGCGGCAGCAGC
TGCCTGAGAAGTACAAAGAGATTTTCTTCGACCAGAGCAAGAACGGCTACGCCGGCTACATTGACGG

CGGAGCCAGCCAGGAAGAGTTCTACAAGTTCATCAAGCCCATCCTGGAAAAGATGGACGGCACCGAG
GAACTGCTCGTGAAGCTGAACAGAGAGGACCTGCTGCGGAAGCAGCGGACCTTCGACAACGGCAGC
ATCCCCCACCAGATCCACCTGGGAGAGCTGCACGCCATTCTGCGGCGGCAGGAAGATTTTTACCCATT
CCTGAAGGACAACCGGGAAAAGATCGAGAAGATCCTGACCTTCCGCATCCCCTACTACGTGGGCCCTC
TGGCCAGGGGAAACAGCAGATTCGCCTGGATGACCAGAAAGAGCGAGGAAACCATCACCCCCTGGA
ACTTCGAGGAAGTGGTGGACAAGGGCGCTTCCGCCCAGAGCTTCATCGAGCGGATGACCAACTTCGA
TAAGAACCTGCCCAACGAGAAGGTGCTGCCCAAGCACAGCCTGCTGTACGAGTACTTCACCGTGTATA
ACGAGCTGACCAAAGTGAAATACGTGACCGAGGGAATGAGAAAGCCCGCCTTCCTGAGCGGCGAGC
AGAAAAAGGCCATCGTGGACCTGCTGTTCAAGACCAACCGGAAAGTGACCGTGAAGCAGCTGAAAG
AGGACTACTTCAAGAAAATCGAGTGCCTGAGCTACGAGACAGAGATCCTGACCGTGGAATACGGCCT
GCTGCCTATCGGCAAGATCGTGGAAAAGCGGATCGAGTGCACCGTGTACAGCGTGGACAACAACGGC
AACATCTACACCCAGCCTGTGGCTCAGTGGCACGACAGAGGCGAGCAAGAGGTGTTCGAGTACTGCC
TGGAAGATGGCAGCCTGATCAGAGCCACCAAGGACCACAAGTTCATGACAGTGGACGGCCAGATGCT
GCCCATCGACGAGATCTTCGAGCGCGAGCTGGACCTGATGAGAGTGGACAACCTGCCTAACTAA

Coding sequence for Int[c]-C-ABE8e.SpG

MIKIATRKYLGKQNVYDIGVERDHNFALKNGFIASNQSGKTILDFLKSDGFANRNFMQLIHDDSLTFKEDI
QKAQVSGQGDSLHEHIANLAGSPAIKKGILQTVKVVDELVKVMGRHKPENIVIEMARENQTTQKGQKNS
RERMKRIEEGIKELGSQILKEHPVENTQLQNEKLYLYYLQNGRDMYVDQELDINRLSDYDVDHIVPQSFL
KDDSIDNKVLTRSDKNRGKSDNVPSEEVVKKMKNYWRQLLNAKLITQRKFDNLTKAERGGLSELDKAGF
IKRQLVETRQITKHVAQILDSRMNTKYDENDKLIREVKVITLKSKLVSDFRKDFQFYKVREINNYHHAHDA
YLNAVVGTALIKKYPKLESEFVYGDYKVYDVRKMIAKSEQEIGKATAKYFFYSNIMNFFKTEITLANGEIR
KRPLIETNGETGEIVWDKGRDFATVRKVLSMPQVNIVKKTEVQTGGFSKESILPKRNSDKLIARKKDWDP
KKYGGFLWPTVAYSVLVVAKVEKGKSKKLKSVKELLGITIMERSSFEKNPIDFLEAKGYKEVKKDLIIKLP
KYSLFELENGRKRMLASAKQLQKGNELALPSKYVNFLYLASHYEKLKGSPEDNEQKQLFVEQHKHYLDE
IIEQISEFSKRVILADANLDKVLSAYNKHRDKPIREQAENIIHLFTLTNLGAPAAFKYFDTTIDRKQYRSTKE
VLDATLIHQSITGLYETRIDLSQLGGDSGGSKRTADGSEFEPKKKRKV

ATGATCAAGATCGCCACACGGAAGTACCTGGGCAAGCAGAACGTGTACGACATCGGCGTGGAACGGG
ACCACAACTTCGCCCTGAAGAACGGCTTTATCGCCAGCAACTGCTTCGACTCCGTGGAAATCTCCGGC
GTGGAAGATCGGTTCAACGCCTCCCTGGGCACATACCACGATCTGCTGAAAATTATCAAGGACAAGGA
CTTCCTGGACAATGAGGAAAACGAGGACATTCTGGAAGATATCGTGCTGACCCTGACACTGTTTGAGG
ACAGAGAGATGATCGAGGAACGGCTGAAAACCTATGCCCACCTGTTCGACGACAAAGTGATGAAGCA
GCTGAAGCGGCGGAGATACACCGGCTGGGGCAGGCTGAGCCGGAAGCTGATCAACGGCATCCGGGA
CAAGCAGTCCGGCAAGACAATCCTGGATTTCCTGAAGTCCGACGGCTTCGCCAACAGAAACTTCATG
CAGCTGATCCACGACGACAGCCTGACCTTTAAAGAGGACATCCAGAAAGCCCAGGTGTCCGGCCAGG
GCGATAGCCTGCACGAGCACATTGCCAATCTGGCCGGCAGCCCCGCCATTAAGAAGGGCATCCTGCAG
ACAGTGAAGGTGGTGGACGAGCTCGTGAAAGTGATGGGCCGGCACAAGCCCGAGAACATCGTGATC
GAAATGGCCAGAGAGAACCAGACCACCCAGAAGGGACAGAAGAACAGCCGCGAGAGAATGAAGCG
GATCGAAGAGGGCATCAAAGAGCTGGGCAGCCAGATCCTGAAAGAACACCCCGTGGAAAACACCCA

GCTGCAGAACGAGAAGCTGTACCTGTACTACCTGCAGAATGGGCGGGATATGTACGTGGACCAGGAA
CTGGACATCAACCGGCTGTCCGACTACGATGTGGACCATATCGTGCCTCAGAGCTTTCTGAAGGACGA
CTCCATCGACAACAAGGTGCTGACCAGAAGCGACAAGAACCGGGGCAAGAGCGACAACGTGCCCTC
CGAAGAGGTCGTGAAGAAGATGAAGAACTACTGGCGGCAGCTGCTGAACGCCAAGCTGATTACCCAG
AGAAAGTTCGACAATCTGACCAAGGCCGAGAGAGGCGGCCTGAGCGAACTGGATAAGGCCGGCTTC
ATCAAGAGACAGCTGGTGGAAACCCGGCAGATCACAAAGCACGTGGCACAGATCCTGGACTCCCGGA
TGAACACTAAGTACGACGAGAATGACAAGCTGATCCGGGAAGTGAAAGTGATCACCCTGAAGTCCAA
GCTGGTGTCCGATTTCCGGAAGGATTTCCAGTTTTACAAAGTGCGCGAGATCAACAACTACCACCACG
CCCACGACGCCTACCTGAACGCCGTCGTGGGAACCGCCCTGATCAAAAAGTACCCTAAGCTGGAAAG
CGAGTTCGTGTACGGCGACTACAAGGTGTACGACGTGCGGAAGATGATCGCCAAGAGCGAGCAGGAA
ATCGGCAAGGCTACCGCCAAGTACTTCTTCTACAGCAACATCATGAACTTTTTCAAGACCGAGATTACC
CTGGCCAACGGCGAGATCCGGAAGCGGCCTCTGATCGAGACAAACGGCGAAACCGGGGAGATCGTG
TGGGATAAGGGCCGGGATTTTGCCACCGTGCGGAAAGTGCTGAGCATGCCCCAAGTGAATATCGTGAA
AAAGACCGAGGTGCAGACAGGCGGCTTCAGCAAAGAGTCTATCCTGCCCAAGAGGAACAGCGATAA
GCTGATCGCCAGAAAGAAGGACTGGGACCCTAAGAAGTACGGCGGCTTCCTGTGGCCCACCGTGGCC
TATTCTGTGCTGGTGGTGGCCAAAGTGGAAAAGGGCAAGTCCAAGAAACTGAAGAGTGTGAAAGAG
CTGCTGGGGATCACCATCATGGAAAGAAGCAGCTTCGAGAAGAATCCCATCGACTTTCTGGAAGCCA
AGGGCTACAAAGAAGTGAAAAAGGACCTGATCATCAAGCTGCCTAAGTACTCCCTGTTCGAGCTGGA
AAACGGCCGGAAGAGAATGCTGGCCTCTGCCAAGCAGCTGCAGAAGGGAAACGAACTGGCCCTGCC
CTCCAAATATGTGAACTTCCTGTACCTGGCCAGCCACTATGAGAAGCTGAAGGGCTCCCCCGAGGATA
ATGAGCAGAAACAGCTGTTTGTGGAACAGCACAAGCACTACCTGGACGAGATCATCGAGCAGATCAG
CGAGTTCTCCAAGAGAGTGATCCTGGCCGACGCTAATCTGGACAAAGTGCTGTCCGCCTACAACAAG
CACCGGGATAAGCCCATCAGAGAGCAGGCCGAGAATATCATCCACCTGTTTACCCTGACCAATCTGGG
AGCCCCTGCCGCCTTCAAGTACTTTGACACCACCATCGACCGGAAGCAGTACAGAAGCACCAAAGAG
GTGCTGGACGCCACCCTGATCCACCAGAGCATCACCGGCCTGTACGAGACACGGATCGACCTGTCTCA
GCTGGGAGGTGACTCTGGCGGCTCAAAAAGAACCGCCGACGGCAGCGAATTCGAGCCCAAGAAGAA
GAGGAAAGTCTAA

Supplementary Methods

Genomic DNA extraction and SURVEYOR assay. Genomic DNA from HEK293 cell line by transient transfection was extracted using the QuickExtract DNA Extraction Solution (Epicentre Biotechnologies). The efficiency of each individual sgRNA was tested by the SURVEYOR nuclease assay (Transgenomics) as described previously[1] using the PCR primers listed in Supplementary Table1.

References

1. Ran, FA, Hsu, PD, Wright, J, Agarwala, V, Scott, DA, and Zhang, F (2013). Genome engineering using the CRISPR-Cas9 system. *Nature Protocols* **8**: 2281-2308.
